# Peptides and Peptide Analogues Targeting Acetylcholinesterase-Associated Mechanisms in Alzheimer’s Disease and Dementias: Evidence Gaps, Design Strategies, and Translational Perspectives

**DOI:** 10.3390/ijms27209094

**Published:** 2026-10-14

**Authors:** Joanna Zdunek, Błażej Grodner

**Affiliations:** Department of Biochemistry and Pharmacogenomics, Medical University of Warsaw, 1 Banacha Str., 02-097 Warsaw, Poland; s086155@student.wum.edu.pl

**Keywords:** Alzheimer’s disease, acetylcholinesterase, peptides, peptidomimetics, amyloid-beta, neuroprotection, cholinergic system, blood–brain barrier, target engagement, multi-target-directed ligands

## Abstract

Alzheimer’s disease and related dementias involve interacting disturbances in amyloid-β, tau, neuroinflammation, oxidative stress, mitochondrial function, synaptic integrity, and cholinergic neurotransmission. Acetylcholinesterase, in addition to terminating cholinergic signalling through acetylcholine hydrolysis, participates in non-catalytic interactions associated with amyloid-β, providing a mechanistic rationale for multi-target therapeutic strategies. This critical narrative review evaluates natural acetylcholinesterase-inhibitory peptides, amyloid-targeting sequences, D-enantiomeric and retro-inverso analogues, endogenous and neuroprotective peptides, and selected peptidomimetic and multifunctional designs. The literature is considered according to an evidence hierarchy that distinguishes direct acetylcholinesterase/peripheral anionic site target engagement from independently demonstrated secondary mechanisms, cellular activity, central nervous system exposure, in vivo pharmacodynamics, and clinical efficacy. The reviewed evidence indicates that peptides and peptide-derived compounds offer substantial molecular recognition and structural adaptability and may, in principle, be engineered to combine selected cholinergic and disease-relevant functions. Importantly, no single candidate reviewed here has experimentally demonstrated all of the proposed cholinergic, anti-amyloid, anti-inflammatory, antioxidant, mitochondrial, and synaptoprotective activities simultaneously. However, neuroprotective, anti-amyloid, or cognitive effects alone do not establish direct acetylcholinesterase modulation, and many proposed multifunctional candidates lack experimental evidence of acetylcholinesterase/peripheral anionic site engagement. Overall, the available literature supports the feasibility of designing multifunctional peptide-based acetylcholinesterase-directed agents, but the evidence remains insufficient to establish a clinically validated class of multifunctional acetylcholinesterase modulators.

## 1. Introduction

Dementia represents a major unmet therapeutic need. Alzheimer’s disease (AD) is the predominant dementia syndrome, while dementia with Lewy bodies (DLB), Parkinson’s disease dementia (PDD), vascular dementia (VaD), frontotemporal dementia (FTD), and mixed pathologies have distinct and overlapping molecular features [1,2]. Cholinergic dysfunction is particularly well established in AD and Lewy body disorders, including DLB and PDD, whereas its magnitude and mechanistic relevance vary across VaD and FTD [1,2,3,4].

Clinically, Alzheimer’s disease typically begins with progressive episodic memory and other cognitive difficulties and may subsequently involve impaired planning, language, visuospatial processing, judgment, personality or behaviour, and loss of independence in daily activities. Dementia is a clinical syndrome characterized by decline in memory, attention, communication, reasoning, judgment, or other cognitive functions sufficient to interfere with everyday life. Dementia with Lewy bodies commonly presents with fluctuating cognition, recurrent visual hallucinations, rapid eye movement (REM)-sleep behaviour disorder, parkinsonism, and autonomic dysfunction, whereas Parkinson’s disease dementia develops cognitive impairment in the setting of established Parkinsonian disease. Vascular dementia may present with slowed information processing, executive dysfunction, gait or focal neurological abnormalities, and a stepwise or heterogeneous course depending on the vascular lesions. Frontotemporal dementia is more often characterized by early behavioural disinhibition or apathy, loss of empathy, compulsive or stereotyped behaviour, executive dysfunction, or progressive language impairment. These clinical phenotypes overlap but are not interchangeable and therefore provide different mechanistic contexts for cholinergic and multi-target interventions [1,2,3,4,5,6,7,8,9].

In AD, degeneration of basal forebrain cholinergic neurons and their projections contributes to cortical and hippocampal cholinergic deficits, which are associated with impairments in attention, learning, and memory [1,2]. In contrast, evidence for cholinesterase inhibitors in VaD and vascular cognitive impairment is more heterogeneous; meta-analytic evidence indicates modest cognitive benefits with some agents, although these effects are generally small and their clinical significance remains uncertain [5]. Evidence in FTD is limited and does not support routine use of cholinesterase inhibitors; moreover, the basal forebrain cholinergic system appears to be substantially more resistant to neurodegeneration in FTD than in AD and diffuse Lewy body disease [1,6,7].

A second conceptual shift arises from structural and cell-biological studies of acetylcholinesterase (AChE). In a seminal experimental study, AChE was shown to accelerate the assembly of amyloid-β (Aβ) peptides into fibrils, whereas this effect was reduced by ligands interacting with the peripheral anionic site (PAS) [10]. Subsequent studies further supported the concept that AChE can interact with Aβ through non-catalytic mechanisms and promote the formation of AChE–Aβ complexes with potentially enhanced neurotoxicity [11,12]. However, these observations should not be interpreted as evidence that every peptide with anti-amyloid or neuroprotective activity is an AChE modulator; direct engagement of AChE, the PAS, or a defined AChE-associated molecular interface must be demonstrated independently.

The clinical context supports interest in multi-target strategies, but it does not by itself validate a particular molecular mechanism. However, their potential advantages must be considered alongside important liabilities, including susceptibility to proteolytic degradation, limited oral bioavailability, uncertain BBB penetration, physicochemical instability and aggregation, and the analytical challenges associated with defining the molecular species responsible for biological activity [13,14,15]. Consequently, the therapeutic potential of multifunctional peptides should be evaluated through an integrated framework encompassing biochemical target engagement, cellular activity, peptide stability and characterization, pharmacokinetic/pharmacodynamic relationships, and efficacy in relevant in vivo disease models rather than inferred from a single phenotypic assay [14,15,16,17].

These limitations help explain why peptide-based approaches remain of interest despite the established symptomatic use of donepezil, rivastigmine, and galantamine in appropriate dementia settings. Peptides offer sequence-defined molecular recognition, the ability to present multiple functional groups within one scaffold, and scope for stereochemical, backbone, cyclization, and conjugation-based optimization. Their liabilities are equally important: proteolytic degradation, limited oral bioavailability, uncertain CNS exposure, manufacturing complexity, and the possibility that chemical modification changes the active molecular species. The rationale for studying peptides is therefore not that they are inherently superior to approved cholinesterase inhibitors, but that they may provide experimentally testable architectures for combining target engagement with a second disease-relevant mechanism [1,2,3,13,14,15,16,17].

The objective of this review is threefold. First, to define an evidence hierarchy for peptide interactions with AChE and AChE-associated pathology, distinguishing direct biochemical evidence from cellular, in vivo, and purely conceptual associations. Second, to critically classify peptide and peptide-analogue candidates according to demonstrated direct AChE/PAS engagement, independently demonstrated secondary mechanisms, and indirect disease-relevant effects. Third, to outline the engineering, central nervous system (CNS) delivery, pharmacokinetic, computational, and translational requirements for converting a proposed multifunctional peptide concept into an experimentally testable candidate. Throughout the review, headings and candidate classifications have been aligned with this evidence framework so that terms such as “direct,” “multifunctional,” and “AChE/PAS-modulating” are reserved for candidates supported by compound-specific evidence.

The global burden of dementia is substantial and increasing. The World Health Organization reported that 57 million people were living with dementia worldwide in 2021 and that nearly 10 million new cases occur each year; Alzheimer’s disease accounts for an estimated 60–70% of dementia cases. In the WHO European Region, approximately 14 million people were living with dementia in 2019, and dementia-related disability-adjusted life years increased from 3.7 million in 2000 to 7.8 million in 2021, with prevalence projected to double by 2030. These data support an increasing population-level burden rather than a declining incidence or prevalence trend [8].

Hypothesis: We hypothesized that peptide and peptide-analogue scaffolds can be rationally designed to combine experimentally demonstrated acetylcholinesterase/peripheral anionic site engagement with an independently validated disease-relevant secondary mechanism. We also hypothesized that the current literature would reveal important evidence gaps preventing classification of such constructs as a clinically validated multifunctional therapeutic class.

## 2. Scope and Literature Review Strategy

This article is a critical narrative review integrating mechanistic, structural, pharmacological, engineering, and translational evidence on peptides and peptide analogues relevant to AChE and cholinergic neurodegeneration in Alzheimer’s disease (AD) and other dementias. Searches were structured around PubMed/MEDLINE, Scopus, Web of Science, and Embase using combinations of the following concepts: (acetylcholinesterase OR AChE) AND (peripheral anionic site OR PAS) AND (peptide* OR peptidomimetic* OR peptide analogue*) AND (Alzheimer* OR dementia OR cholinergic OR amyloid-β OR tau OR neuroprotect*). Candidate-specific searches were additionally performed using individual peptide names and sequence identifiers. Because the original narrative-review workflow did not prospectively archive database run dates or database-specific query logs, exact historical search dates cannot be reconstructed retrospectively and are therefore not inferred here. To improve reproducibility, the conceptual master search string and the eligibility framework are now stated explicitly, while database-specific syntax and indexing terms may vary. Studies were eligible when they reported mechanistic, biochemical, structural, pharmacological, cellular, animal, pharmacokinetic, or clinical evidence relevant to the review question. Studies were prioritized when they provided compound-specific quantitative activity, direct target-engagement evidence, defined experimental conditions, or independently validated secondary mechanisms. Purely conceptual papers, non-peptide compounds, and reports without sufficient relevance to AChE/cholinergic or disease-associated peptide mechanisms were not used as primary evidence. This was a narrative review rather than a systematic review; therefore, no protocol-driven screening, duplicate-independent study selection, or formal meta-analytic risk-of-bias procedure was undertaken [18,19].

Because the evidence base is heterogeneous, the review uses four principal evidence categories. Direct AChE evidence indicates that an interaction with AChE or its PAS has been experimentally demonstrated, preferably using direct biochemical, biophysical, structural, or binding approaches. Clinical evidence requires human intervention data with clearly defined endpoints and outcome measures. This hierarchy is intended to distinguish molecular target engagement from downstream biological effects and, importantly, from clinical efficacy; these levels of evidence should not be considered interchangeable [18,19] (Table 1).

The distinction between these categories is particularly important for peptide-based candidates because a favourable cellular or behavioural phenotype may arise from multiple molecular mechanisms and does not, by itself, establish the identity of the molecular target [26,27]. This distinction is particularly relevant to multifunctional approaches in AD, in which a single candidate may simultaneously display cholinesterase inhibition, anti-amyloid, antioxidant, anti-inflammatory, or neuroprotective activities without establishing that all observed effects arise from direct interaction with AChE [10,27].

Throughout the review, a “multifunctional AChE-related peptide” is defined conservatively. This criterion is consistent with the broader concept of multitarget-directed agents, in which activity against more than one biologically relevant target or pathway should be experimentally substantiated rather than inferred from a single downstream phenotype [28,29]. This definition is therefore intended to distinguish molecular target engagement from downstream pharmacological or phenotypic effects and to avoid assigning a molecular mechanism on the basis of phenotype alone.

For operational purposes, a candidate is classified as a multifunctional AChE-related peptide only when (a) direct AChE/PAS interaction or modulation is experimentally demonstrated and (b) at least one additional disease-relevant mechanism is independently demonstrated in the same molecular entity. Candidates with only one demonstrated function plus a second predicted or phenotypic function are classified as design hypotheses, not validated multifunctional modulators. The candidate matrices in Tables in Section 7 and Section 10 apply this rule at the candidate level.

Peptides with anti-amyloid or neuroprotective activity but without direct AChE/PAS evidence are classified as AChE-associated or indirect candidates, rather than as validated direct AChE modulators. Demonstration of target engagement should therefore be complemented by mechanistic studies linking AChE interaction to the relevant downstream phenotype and, where possible, by orthogonal approaches that establish causality between target modulation and biological activity [20,28,29,30].

Accordingly, the review distinguishes (i) demonstrated AChE/PAS engagement, (ii) independently demonstrated secondary mechanisms, and (iii) downstream biological or clinical effects. This distinction is central to the interpretation of the literature and to the classification of candidate peptides throughout the review, as evidence of target engagement, mechanistic activity, and therapeutic efficacy represents related but non-equivalent levels of evidence.

## 3. AChE as a Multifunctional Target in Alzheimer’s Disease and Other Dementias

### 3.1. Cholinergic Dysfunction Across Dementia Subtypes

Cholinergic dysfunction in AD represents the clearest clinical context for AChE-targeted therapy. Beyond AD, substantial cholinergic deficits have also been documented in dementia with Lewy bodies (DLB) and Parkinson’s disease dementia (PDD), with evidence from neuropathological and neurochemical studies indicating that cholinergic dysfunction may be particularly pronounced in Lewy body disorders [1,4]. This neuropathological and neurochemical evidence is consistent with the clinical use of cholinesterase inhibitors in DLB and PDD, for which clinical evidence supports symptomatic benefits, particularly in cognitive and neuropsychiatric domains [1,3].

By contrast, the role of cholinergic dysfunction in vascular dementia (VaD) appears more heterogeneous. Cholinergic impairment may result from ischemic damage to basal forebrain cholinergic nuclei and their projections, whereas the clinical evidence supporting cholinesterase inhibitor therapy in VaD is more variable than in AD and may be influenced by the frequent coexistence of AD-related pathology [2,31]. In frontotemporal dementia (FTD), evidence for a primary cholinergic deficit is substantially weaker, and cholinesterase inhibitors have not demonstrated the therapeutic rationale established in AD or Lewy body dementias [3,24]. Moreover, some clinical studies have reported worsening of behavioural symptoms in patients with FTD treated with cholinesterase inhibitors, including increased disinhibition and compulsive behaviours with donepezil [32]. Thus, AChE can be considered a clinically relevant cholinergic node across several dementia syndromes, but the strength and biological significance of cholinergic involvement are disease-dependent.

A further distinction is required regarding the non-catalytic functions of AChE. Experimental studies have demonstrated that AChE can interact with Aβ and promote amyloid fibril formation, with evidence implicating molecular regions associated with the PAS in this interaction [10,11,22,33]. Subsequent studies further showed that AChE–Aβ complexes can alter the biochemical properties of AChE and increase the neurotoxicity of amyloid aggregates, supporting a potential role for AChE in amyloid-associated pathology [10,12]. These findings provide a mechanistic rationale for investigating AChE/PAS as a component of amyloid-associated processes, particularly in AD.

This distinction has direct implications for peptide design. A peptide with experimentally demonstrated direct AChE inhibitory activity may have symptomatic therapeutic relevance in disorders characterized by substantial cholinergic dysfunction, although its clinical relevance would depend on appropriate pharmacological validation and target engagement [1,2,3,20]. In contrast, a peptide designed to interfere specifically with the AChE–Aβ interface has a more selective mechanistic rationale for amyloid-associated pathology, particularly in AD, based on experimental evidence that AChE can promote Aβ aggregation and modulate the properties and neurotoxicity of AChE–Aβ complexes [10,11,12,22]. Extending this latter mechanism to DLB, PDD, VaD, or FTD should therefore be considered a hypothesis rather than an established therapeutic principle unless the relevant AChE-associated pathological interaction, biological effect, and peptide target engagement have been experimentally demonstrated [20,21,23].

The disease context must consequently be specified whenever a peptide is described as “AChE-related.” Demonstration of AChE inhibition establishes a pharmacological interaction with the enzyme, but does not by itself establish disease modification; conversely, an anti-amyloid or neuroprotective phenotype does not establish direct AChE/PAS engagement in the absence of appropriate target-engagement experiments [20,21,23]. This distinction is central to the evidence hierarchy adopted in the present review, in which biochemical activity, target engagement, cellular or phenotypic effects, and disease-relevant outcomes are considered distinct levels of evidence.

**Figure 1:**
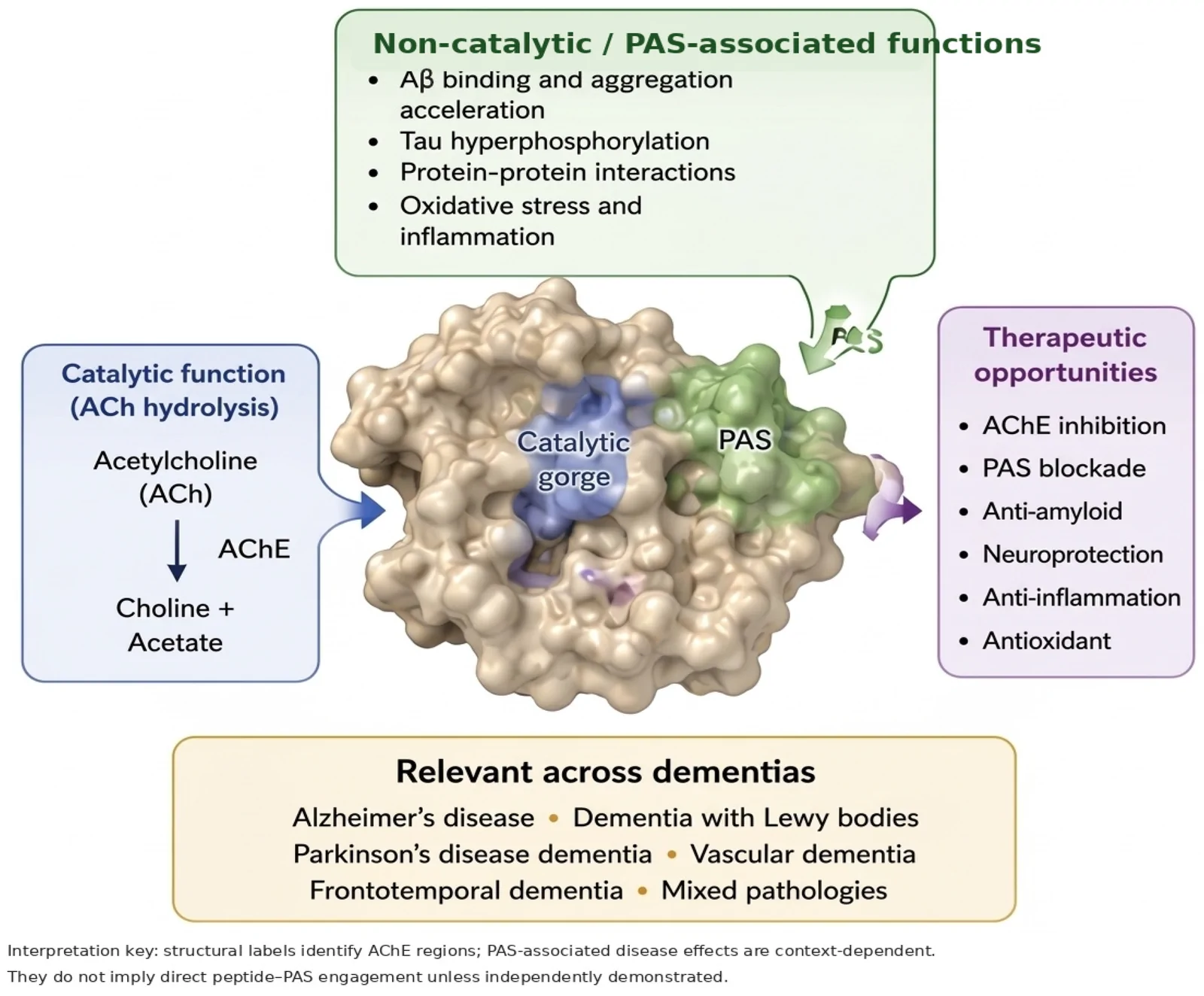
AChE as a multifunctional target: catalytic gorge, PAS, non-catalytic functions, and disease-context-dependent therapeutic opportunities across dementia subtypes. PAS-associated effects on Aβ/tau or inflammatory pathways are presented as mechanistic associations rather than evidence of direct peptide–PAS engagement unless supported by compound-specific biochemical or structural data. The annotation distinguishes established structural regions from context-dependent disease associations; arrows do not imply that every listed downstream effect is PAS-mediated.

Figure 1 summarizes the mechanistic or translational relationships described in this section.

### 3.2. Structural Basis for Multifunctional AChE Peptide Design

The structural biology of AChE provides a mechanistic basis for exploring multifunctional ligand design. The catalytic active site is located at the bottom of a deep and narrow gorge lined predominantly by aromatic residues, whereas the PAS is positioned near the entrance of the gorge [34,35]. This architecture creates spatially distinct regions that can be targeted independently or simultaneously.

The three-dimensional organization of the AChE gorge is particularly relevant to the design of ligands containing multiple recognition elements. Structural and biochemical studies have demonstrated that ligands can interact with both catalytic and peripheral regions of AChE, providing a precedent for dual-site recognition and bivalent ligand design [34,35,36]. Peptide and peptidomimetic scaffolds may, in principle, exploit multiple contact points and conformational adaptability to engage extended or spatially separated molecular surfaces.

The PAS is particularly relevant to multifunctional AChE peptide design because it participates in biological interactions beyond acetylcholine hydrolysis. Structural and biochemical studies have identified the PAS as an important region for interactions with diverse ligands and protein partners [33,34]. Most notably, AChE has been experimentally shown to interact with Aβ and to accelerate Aβ fibril formation, with the peripheral region of the enzyme implicated in this process [11,22]. Subsequent studies provided further evidence that AChE can form stable complexes with Aβ and that these complexes may alter the biochemical properties of AChE and increase the neurotoxicity of amyloid aggregates [10,12]. Collectively, these findings provide a mechanistic rationale for investigating PAS-directed ligands as potential modulators of AChE-associated amyloid biology, while not by themselves establishing the clinical validity of this mechanism as a therapeutic target.

Accordingly, the AChE–Aβ interaction can be viewed as a potential mechanistic interface linking the classical cholinergic function of AChE with a non-catalytic, amyloid-related activity [10,11,12,22]. This concept provides a rationale for multifunctional peptide strategies in which PAS recognition is combined with an independently validated amyloid-directed or neuroprotective function. The multifunctional biology of AChE is therefore supported at the structural and experimental mechanistic levels, whereas the therapeutic value of exploiting these functions with multifunctional peptides remains to be established through direct experimental validation, including appropriate target-engagement and disease-relevant efficacy studies [20,21,23].

**Figure 2:**
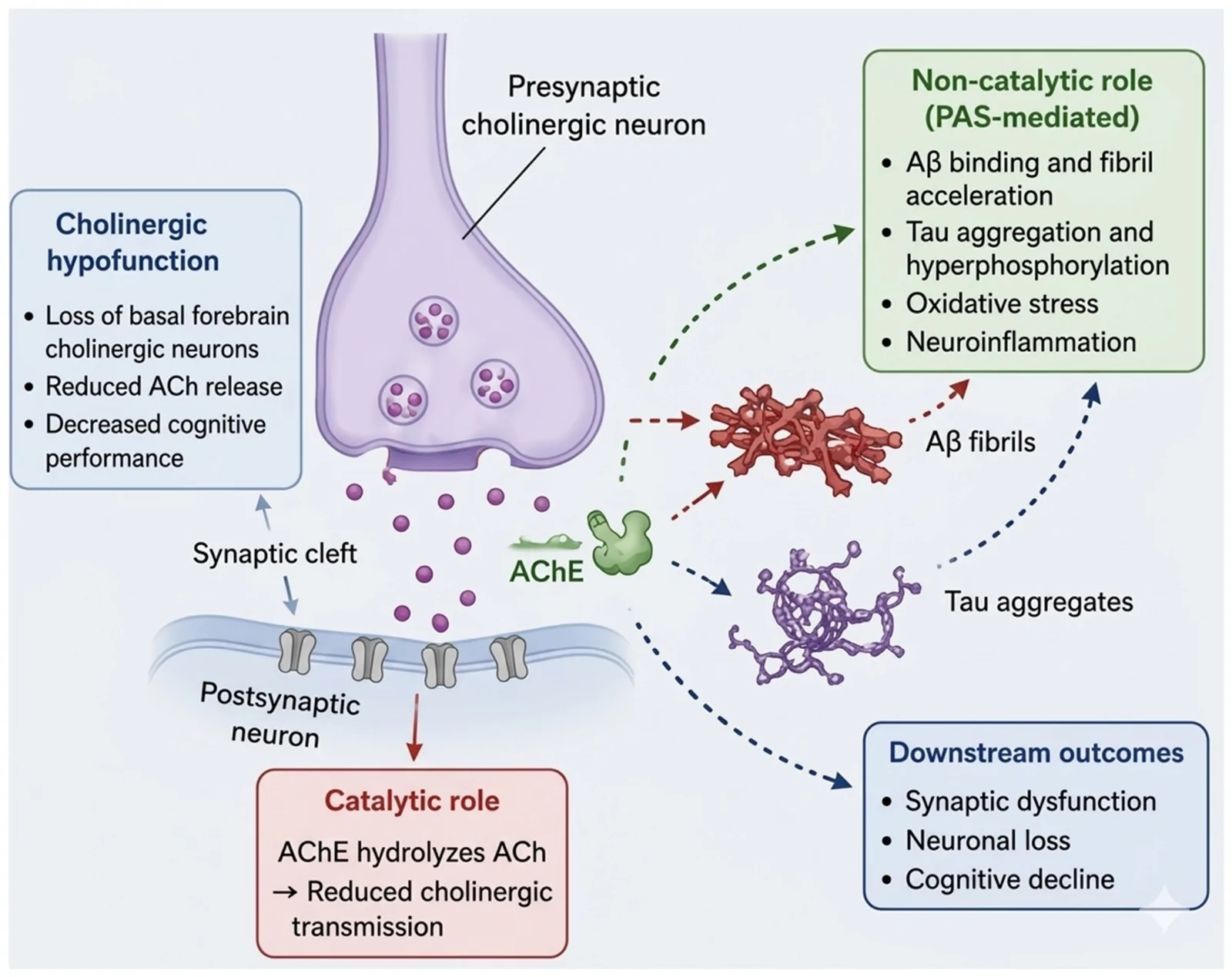
Mechanistic links between cholinergic dysfunction, AChE/PAS, amyloid-β (Aβ), tau-related pathology, inflammation, and downstream neurodegenerative outcomes. The relationships shown are disease- and mechanism-dependent; PAS-associated tau or inflammatory effects should not be interpreted as universal consequences of AChE inhibition or as evidence of direct peptide target engagement. Original schematic prepared for this manuscript.

Figure 2 summarizes the mechanistic or translational relationships described in this section.

AChE can accelerate amyloid fibril assembly through interactions involving its PAS, making the PAS a mechanistically relevant interface in amyloid-associated AD pathology [11,22]. Experimental studies indicate that AChE can interact with Aβ and promote its aggregation into fibrillar assemblies, while interactions involving the peripheral region of the enzyme can modulate this process [10,11,12,22]. PAS-directed engagement may therefore complement catalytic-site modulation in a multifunctional design, particularly when simultaneous modulation of cholinergic and amyloid-related pathways is intended.

The AChE–Aβ framework may also be relevant to mixed-pathology cohorts in which amyloid co-pathology contributes to overall disease burden. Mixed AD and Lewy body pathology is common in older individuals with dementia, and concomitant amyloid pathology may influence the clinical and biological phenotype [1,4]. Nevertheless, extension of an AChE–Aβ mechanism to DLB or PDD should be presented as a conditional hypothesis rather than an established disease mechanism.

In VaD and FTD, the rationale for an AChE–Aβ mechanism is even less direct. Vascular cognitive impairment is biologically heterogeneous and may coexist with AD-type amyloid pathology, but vascular injury itself does not establish an AChE–Aβ mechanism [2,31]. Similarly, the molecular pathology of FTD is predominantly associated with proteinopathies involving tau, TAR DNA-binding protein 43 (TDP-43), or fused in sarcoma (FUS) rather than a primary Aβ-driven process [3,24]. Accordingly, evidence obtained in AD should not be generalized to VaD or FTD without disease-specific demonstration of the relevant pathology, molecular interaction, and peptide target engagement [20,21,23].

## 4. Peptides and Peptide Analogues with Direct or Putative AChE Modulatory Evidence

**Figure 3:**
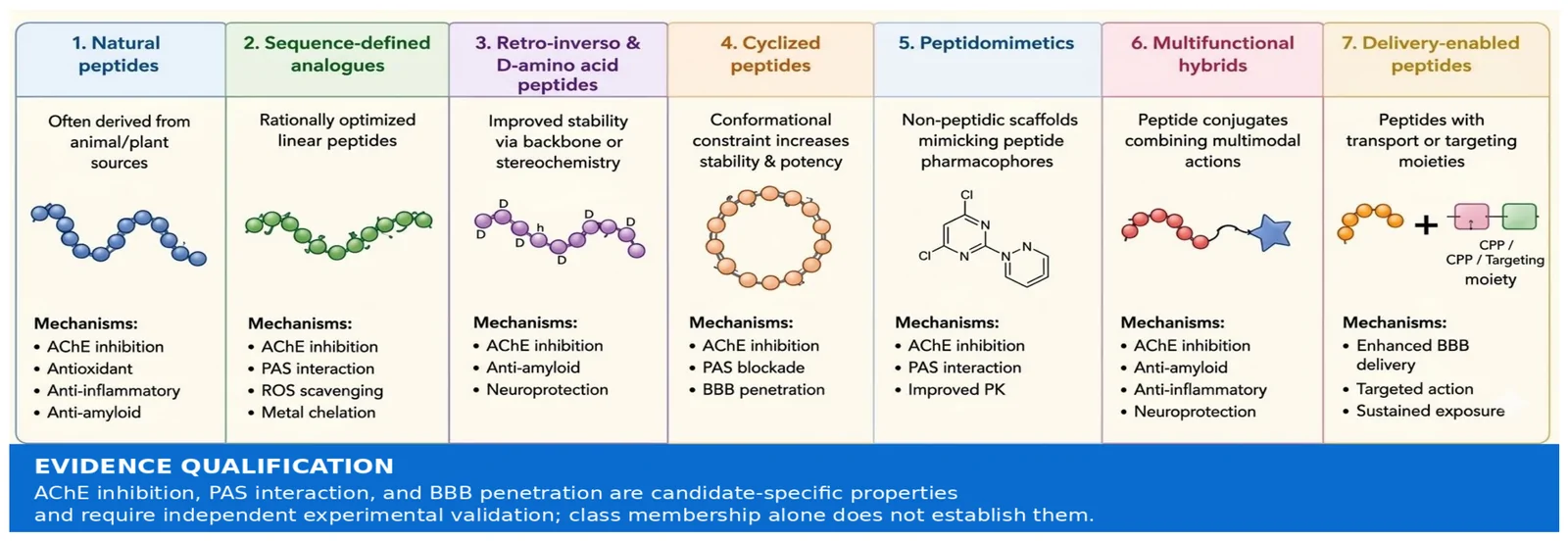
Major classes of peptide and peptide-analogue strategies relevant to AChE modulation. The class-level architecture shown here represents design space rather than uniform target engagement: direct AChE inhibition, PAS interaction, and improved BBB penetration are candidate-specific properties and require independent experimental support. Delivery-enabled constructs should therefore not be interpreted as established AChE modulators solely on the basis of their peptide class or transport strategy.

Figure 3 summarizes the mechanistic or translational relationships described in this section.

Natural peptide-derived AChE inhibitors provide the most relevant starting point for a target-centred discussion because their activity can be quantified directly at the enzyme level. However, the published evidence is heterogeneous: some studies report only percentage inhibition at a single concentration, whereas a smaller subset provides concentration–response curves, kinetic constants, and structural hypotheses for binding. Accordingly, the present review gives priority to sequence-defined peptides for which half-maximal inhibitory concentration (IC_50_) and, where available, inhibition constant (K_i_) and an experimentally or computationally supported binding mode can be stated.

For peptide inhibitors, IC_50_ values must be interpreted together with enzyme source, substrate concentration, assay format, peptide purity, concentration range, and pre-incubation conditions. IC_50_ is an assay-dependent descriptor and is not equivalent to a dissociation constant; K_i_ is preferable for comparing inhibition mechanisms when a validated kinetic model is available. In addition, AChE potency should be accompanied by butyrylcholinesterase (BChE) profiling and, where PAS involvement is claimed, an orthogonal binding or structural experiment. The current literature contains relatively few peptide studies that meet all of these criteria.

Chemical optimisation should therefore be discussed as a sequence–structure–activity problem. Changes in residue identity, length, charge, hydrophobicity, stereochemistry, cyclisation or backbone chemistry may alter both AChE affinity and the relative contribution of catalytic active site (CAS) versus peripheral anionic site (PAS) contacts. A modified peptide should consequently be evaluated against its parent sequence under matched conditions, rather than assuming that improved stability or predicted docking automatically preserves AChE pharmacology.

On the basis of the quantitative studies identified here, the strongest direct peptide evidence currently concerns reversible enzyme inhibition with CAS/PAS binding hypotheses rather than experimentally resolved peptide–PAS binding kinetics. Docking to PAS residues supports a structural hypothesis, but does not substitute for surface plasmon resonance (SPR), isothermal titration calorimetry (ITC), nuclear magnetic resonance (NMR), mutational analysis, crystallography/cryogenic electron microscopy (cryo-EM), or another orthogonal target-engagement method.

Experimentally distinguishing catalytic-site from PAS interaction requires more than docking. Competitive versus non-competitive kinetic behaviour can provide an initial indication of whether inhibition is compatible with occupation of the catalytic gorge, but kinetic mode alone does not prove a binding site. Stronger assignment can be obtained by competition with site-selective reference ligands, site-directed mutagenesis of PAS residues, direct binding measurements using orthogonal methods such as SPR, ITC or NMR, and structural methods that resolve the peptide position. For a peptide claimed to engage both sites, matched kinetic, competition, mutational and structural data should be interpreted together; docking should remain hypothesis-generating rather than being treated as direct PAS evidence [33,34,35,37].

### Quantitative AChE Inhibition and Structure–Activity Relationships

A useful quantitative example is provided by five short peptides identified from yellow field pea protein hydrolysates: QSQS, LQHNA, SQSRS, ETRSQ, and PQDER. Their IC_50_ values were narrowly distributed (1.53–1.60 μg/mL; 2.78–4.25 μM), but their kinetic behaviour differed substantially. QSQS showed mixed inhibition (K_i_ 0.43 mM), LQHNA showed non-competitive inhibition (K_i_ 0.16 mM), SQSRS showed uncompetitive inhibition (K_i_ 0.02 mM), while ETRSQ and PQDER showed non-competitive inhibition (K_i_ 0.06 and 0.08 mM, respectively). Thus, similar IC_50_ values do not imply an identical molecular mechanism.

The sequence-level comparison suggests a preliminary structure–activity relationship (SAR) rather than a validated pharmacophore. All five peptides contain glutamine, and the study identified the presence of glutamine at the penultimate N-terminal position as a feature associated with stronger predicted AChE affinity. Docking placed the peptides in the region between PAS and CAS and identified contacts with PAS residues Tyr72, Asp74, Tyr124, Trp286 and Tyr341 together with one or more catalytic-triad residues. The strongest reported docking scores were observed for LQHNA, SQSRS and PQDER. These results support a bimodal CAS/PAS binding hypothesis, but because the PAS assignment was based on docking rather than a direct binding experiment, it should be presented as a testable structural model rather than definitive PAS target engagement.

A second sequence-defined example is the albumin-derived pentapeptide KLPGF. A review of natural AChE-targeting peptides reports an IC_50_ of 1.09 μM, while the primary study demonstrated 61.23 ± 4.73% AChE inhibition at 50 μg/mL and molecular docking predicted contacts with catalytic and peripheral/allosteric regions of AChE. The available evidence therefore supports direct enzymatic inhibition and a plausible extended-gorge interaction, but does not establish a quantitative AChE–PAS affinity constant. Collectively, these studies illustrate the current state of the field: biochemical potency is measurable for several short peptides, whereas rigorous kinetic and orthogonal target-engagement data remain sparse.

For the pivotal biochemical study of pea-derived peptides, AChE was purified from electric eel and activity was measured using an Ellman assay in 0.1 M phosphate buffer (pH 7.4), with AChE at 5 U/mL for IC_50_ determination; kinetic experiments used 0.5 U/mL AChE, four substrate concentrations (0.156–1.25 mM), peptide concentrations of 10 and 50 μg/mL, 37 °C incubation, and absorbance at 405 nm. IC_50_ values were obtained by nonlinear regression, whereas inhibition modes and K_i_ values were derived from Lineweaver–Burk analyses. The kinetic data were reported as mean ± SD from three independent measurements (n = 3), with significance testing at *p* < 0.05. The same study also used fluorescence and circular-dichroism measurements and molecular docking as orthogonal, but not equivalent, evidence of peptide–AChE interaction [38]. The quantitative results are summarized in Table 2.

Table 2 shows that similar inhibitory concentrations can coexist with different kinetic modes, while PAS assignments remain largely docking-based; these findings support direct biochemical AChE inhibition but also identify a gap between enzyme activity and experimentally resolved PAS engagement.

## 5. Peptides at the AChE–Aβ/PAS Interface: Evidence and Design Hypotheses

Table 3 Presents quantitative biochemical and structure–activity data for representative peptide AChE inhibitors. Pea-peptide values are from Asen et al.; *IC_50_ value reported in the 2018 review by Prasasty et al.; NR, not reported. Docking-derived CAS/PAS assignments are not equivalent to direct binding measurements.

### 5.1. Aβ-Derived Recognition Sequences

KLVFF (Lys-Leu-Val-Phe-Phe), corresponding to residues 16–20 of amyloid-β, is a prototypical recognition sequence for peptide-based interference with Aβ self-association and fibril formation. Tjernberg et al. demonstrated that peptides incorporating KLVFF can bind full-length Aβ and inhibit its assembly into amyloid fibrils, establishing Aβ(16–20) as an important self-recognition motif [39]. Subsequent studies confirmed the utility of KLVFF-derived sequences and their derivatives as templates for the development of Aβ aggregation inhibitors, including protease-stable and chemically modified analogues [40]. KLVFF-derived constructs therefore provide evidence for amyloid recognition and modulation of Aβ aggregation, rather than direct AChE inhibition. The evidence status of the principal peptide scaffolds discussed in this section is summarized in Table 3.

Their relevance to the AChE–Aβ interface is mechanistically plausible because AChE can participate in Aβ assembly and promote amyloid fibril formation through interactions involving its peripheral region [10,11,12,22]. However, binding to Aβ itself is not equivalent to binding to AChE or to the AChE–Aβ complex. Unless direct interaction with AChE, the PAS, or a defined AChE–Aβ complex has been experimentally demonstrated, such constructs should be classified as Aβ-directed or AChE-associated candidates, rather than as direct AChE modulators [20,21,23].

The therapeutic rationale for Aβ-directed peptides is strongest when the pathological species and underlying mechanism are explicitly defined. A convincing multifunctional construct would therefore require independent evidence for both components of the proposed mechanism: experimentally demonstrated Aβ-related activity and experimentally demonstrated AChE/PAS-related activity. Ideally, these claims should be supported by orthogonal biochemical, biophysical, or structural assays, with direct target-engagement measurements used where appropriate to establish the molecular interaction [20,21,23].

### 5.2. Retro-Inverso and D-Amino-Acid Analogues

Retro-inverso analogues such as RI-OR2 illustrate how amyloid-recognition sequences can be modified to increase resistance to proteolytic degradation while retaining anti-amyloid activity. RI-OR2 was designed from the OR2 sequence and was reported to resist degradation in human plasma and brain extracts, bind Aβ42, and inhibit the formation of Aβ oligomers and fibrils in vitro [41]. These findings establish RI-OR2 as a proteolytically stable Aβ-directed scaffold, but do not provide evidence of direct AChE/PAS engagement.

Similarly, D-amino-acid peptides such as D3 and RD2 illustrate the use of stereochemical modification to obtain proteolytically resistant anti-amyloid scaffolds. Experimental studies have shown that D3 and RD2 bind Aβ and interfere with β-structure formation and amyloid aggregation [42,43]. RD2 has additionally been investigated as an all-D peptide, with experimental evidence indicating that its interaction with Aβ42 can delay secondary-structure conversion and fibril formation [44]. Their principal evidence therefore concerns Aβ recognition and amyloid-related outcomes rather than direct modulation of AChE.

These scaffolds may provide useful starting points for future bifunctional designs in which an independently validated AChE/PAS-binding element is incorporated. Such an engineered construct should, however, be regarded as a new molecular entity requiring direct demonstration of the newly introduced AChE/PAS interaction.

### 5.3. Multifunctional Peptide Design Combining AChE and Amyloid Engagement

The multi-target-directed ligand (MTDL) framework provides a rational strategy for designing single molecular entities capable of engaging multiple disease-relevant targets or pathological pathways. In AD, this concept has been extensively explored for compounds combining cholinesterase inhibition with anti-amyloid activity and other pharmacological functions [28,29]. Peptide scaffolds may, in principle, be attractive for such approaches because their sequence architecture can accommodate multiple recognition elements within a single molecular framework.

However, multifunctionality must be balanced against molecular size, conformational stability, proteolytic susceptibility, pharmacokinetic properties, tissue distribution, and CNS exposure [13,14,15]. The addition of multiple functional elements may broaden the intended pharmacology but can also alter affinity, selectivity, physicochemical properties, metabolic stability, manufacturing requirements, and the interpretation of in vivo phenotypes. Consequently, multifunctionality should be demonstrated experimentally rather than inferred from the intended architecture of a construct.

The most defensible multifunctional AChE candidates are those in which each claimed function is independently validated—for example, simultaneous PAS engagement and Aβ recognition, or direct AChE modulation combined with a separately demonstrated neuroprotective mechanism [10,11,12,20,21,22,23]. A peptide that combines two unvalidated mechanisms should not be classified as a multifunctional AChE modulator; rather, it represents a multifunctional design hypothesis requiring experimental validation.

### 5.4. Multifunctional Modulators of AChE in DLB and Mixed Dementia

DLB is characterized by α-synuclein pathology together with prominent cholinergic dysfunction, providing a biological rationale for cholinergic intervention [1,4]. A future multifunctional peptide could, in principle, combine AChE/cholinergic modulation with α-synuclein recognition or aggregation modulation. However, within the scope of the present review, this should be regarded as a design hypothesis, rather than as an established AChE–α-synuclein peptide strategy.

Mixed pathology is common in older individuals with dementia, and concomitant amyloid, Lewy body, tau, and vascular lesions may contribute to individual clinical phenotypes [1,4]. Nevertheless, the presence of multiple pathologies does not justify assuming that a single peptide can therapeutically address amyloid, α-synuclein, tau, vascular injury, and cholinergic dysfunction simultaneously. A multi-target scaffold should instead be developed around a defined clinical and mechanistic hypothesis, with each proposed target interaction independently validated [20,21,23,28,29]. This requirement is particularly important for peptide therapeutics, for which increasing molecular complexity may simultaneously increase mechanistic uncertainty and pharmacological liabilities, including effects on stability, distribution, and CNS exposure [13,14,15].

## 6. Neuropeptides as Indirectly Cholinergic-Relevant and Neuroprotective Peptides

Endogenous and synthetic neuropeptides represent a heterogeneous group of signalling molecules with pleiotropic effects on neuronal survival, synaptic plasticity, neuroinflammation, intracellular stress responses, and protein homeostasis. Some of these peptides have been investigated in models of Alzheimer’s disease (AD) and other neurodegenerative disorders, where they may influence cholinergic function indirectly or counteract pathological processes associated with cognitive decline. In the absence of experimental evidence demonstrating direct interaction with AChE or a reproducible alteration in AChE activity, expression, turnover, or function, these peptides should not be classified as AChE modulators.

Pituitary adenylate cyclase-activating polypeptide (PACAP) and vasoactive intestinal peptide (VIP) are primarily receptor-mediated signalling peptides [45]; neuropeptide Y (NPY), galanin and Substance P act through their respective neuropeptide receptors and can influence cholinergic circuits or inflammatory responses without requiring direct AChE binding. Humanin is mainly associated with neuronal-survival and mitochondrial signalling and has been investigated as a neuroprotective factor in AD-related models [46,47,48]. Semax has anti-amyloid and metal-chelating properties [49,50,51,52], whereas GK-2 is an nerve growth factor (NGF)-loop mimetic [53]. Davunetide (NAPVSIPQ) is principally a microtubule/tau-directed neuroprotective peptide [54,55,56]. Cerebrolysin is a heterogeneous peptidergic preparation rather than a single molecular entity and therefore cannot be assigned a sequence-defined AChE mechanism [57,58,59,60,61,62].

PACAP and VIP are primarily receptor-mediated signalling peptides; NPY, galanin and Substance P act through their respective neuropeptide receptors and can influence cholinergic circuits or inflammatory responses without requiring direct AChE binding. Humanin is mainly associated with neuronal-survival and mitochondrial signalling. Semax has anti-amyloid and metal-chelating properties, whereas GK-2 is an NGF-loop mimetic. Davunetide (NAPVSIPQ) is principally a microtubule/tau-directed neuroprotective peptide. Cerebrolysin is a heterogeneous peptidergic preparation rather than a single molecular entity and therefore cannot be assigned a sequence-defined AChE mechanism.

Neuropeptides are retained in this review only as an indirect comparator group. PACAP, VIP, NPY, galanin, Substance P, Humanin, Cerebrolysin, davunetide, Semax and GK-2 have been investigated for neuroprotection, trophic signalling, inflammation, amyloid-related phenotypes, mitochondrial effects or cognition, but the evidence summarized in the cited studies does not establish direct AChE/PAS engagement. Their inclusion therefore provides context for distinguishing cholinergic-network or neuroprotective activity from enzyme-directed pharmacology, rather than evidence that these molecules are AChE inhibitors. The neuropeptides and preparations are summarized in Table 4.

## 7. Translational Lessons from Neuroprotective and Neurotrophic Peptides

Neuroprotective and neurotrophic peptides are retained here primarily to define the translational boundary of the AChE-centred strategy. Their relevance is not established AChE modulation, but the extent to which independently defined neuroprotective mechanisms can progress through preclinical and clinical development without direct AChE/PAS engagement.

The detailed molecular classification of PACAP, VIP, NPY, galanin, Substance P, Humanin, Cerebrolysin, davunetide, Semax and GK-2 is provided in Section 6. This section therefore avoids repeating their mechanisms and instead focuses on what their development histories imply for multifunctional AChE peptide design. The translational characteristics of the principal peptide modulators are summarized in Table 5.

**Table 5:** Peptide modulators and translational lessons relevant to multifunctional AChE design.

Peptide/Modulator	Primary Mechanism	Translational Evidence	Implication for Multifunctional AChE Design
Cerebrolysin	Heterogeneous neurotrophic/peptidergic activity [57,58,59,60,61,62]	Randomized studies in AD and vascular dementia; heterogeneous evidence	Clinical feasibility does not establish a molecular AChE mechanism
Davunetide	Microtubule/tau-related neuroprotection [54,55,56]	Clinical development; phase 2/3 PSP study did not meet primary endpoints [56]	Mechanistic plausibility requires CNS exposure and target-engagement confirmation
Semax	Anti-amyloid, metal-chelating and redox-related effects [49,50,51,52]	Primarily preclinical evidence	Potential secondary mechanism, but AChE/PAS engagement must be demonstrated independently
GK-2	NGF-loop mimetic neurotrophic activity [53]	Preclinical AD models [53]	Provides a neurotrophic module rather than evidence of AChE modulation

### 7.1. Cerebrolysin and the Limits of Multimodal Clinical Evidence

Cerebrolysin provides a clinically developed peptide-derived preparation, but its heterogeneous composition prevents assignment of a sequence-defined AChE mechanism. Randomized studies in AD and vascular dementia have reported cognitive or global benefits in some settings, while systematic evidence remains limited by heterogeneity and risk of bias [57,60,61,62].

The translational lesson is therefore not that a multimodal peptide preparation validates multifunctional AChE modulation. Rather, Cerebrolysin illustrates that clinical feasibility, biological activity and molecular target engagement are separate evidence levels. A clinically observed phenotype cannot retrospectively establish AChE/PAS engagement when the active molecular species and molecular mechanism are not defined.

### 7.2. Davunetide as a Test of Mechanism-to-Clinic Translation

Davunetide provides a more molecularly defined example. Its principal rationale concerns microtubule and tau-related neuroprotection rather than AChE inhibition [54,55,56]. In clinical development, the prespecified endpoints of the phase 2/3 progressive supranuclear palsy (PSP) trial were not significantly improved, while exploratory subgroup findings from the earlier amnestic mild cognitive impairment (aMCI) study remain hypothesis-generating rather than confirmatory [53,56].

This history is directly relevant to multifunctional AChE peptide development. A well-defined mechanism does not guarantee clinical efficacy: adequate CNS exposure, target engagement, dose–response relationships, disease-stage matching, biomarker confirmation and appropriate clinical endpoints must also be established. The existence of a second validated neuroprotective mechanism should therefore not be presented as evidence that adding AChE activity will necessarily improve translation.

### 7.3. Translational Implication for Multifunctional AChE Peptides

The available clinical experience therefore sets a high evidentiary bar for future multifunctional constructs. The relevant question is not whether several biological activities can be detected, but whether a single intact molecular species reaches the CNS, engages AChE/PAS at pharmacologically relevant exposure, independently engages its second target or pathway, and produces a disease-relevant pharmacodynamic effect that can be linked to clinical benefit [20,21,23,75,76].

A multifunctional design that fails at any of these steps may still be useful as a mechanistic research tool, but it should not be described as clinically validated. This interpretation also explains why the present review distinguishes direct AChE candidates from indirect neuroprotective comparators rather than treating all pleiotropic peptides as members of one therapeutic class.

## 8. Molecular Engineering of Peptides as Multifunctional Modulators of AChE

**Figure 4:**
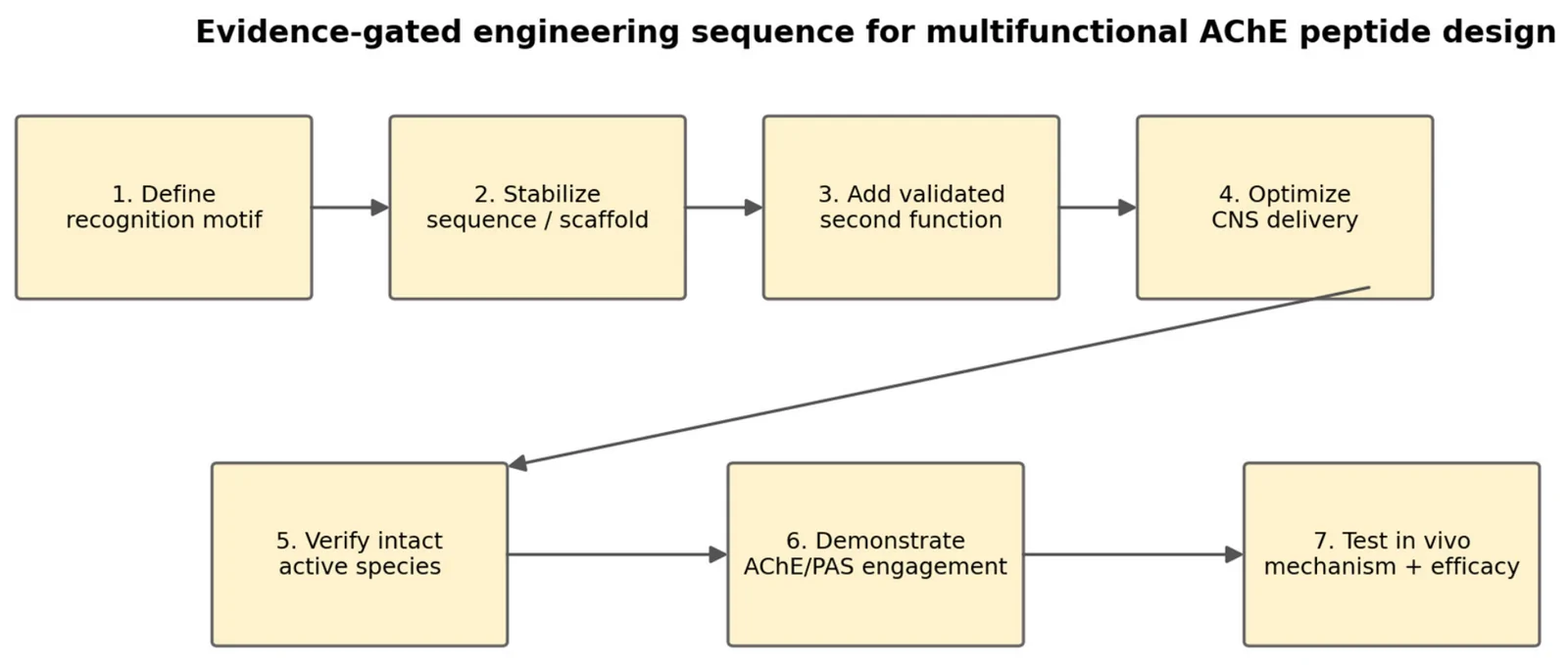
Evidence-gated engineering sequence for multifunctional AChE peptide design. The diagram structures the engineering strategies discussed in Section 8; each modification requires re-validation of molecular recognition, stability, CNS exposure, and target engagement.

The development of peptide-based multifunctional modulators of AChE requires simultaneous optimization of molecular recognition, proteolytic stability, pharmacokinetics, cellular activity, and central nervous system (CNS) exposure. Peptide engineering should therefore be regarded as an integral component of drug design rather than as a secondary optimization step [41,77,78]. Importantly, most strategies discussed below have been established in the broader field of therapeutic peptide and amyloid-targeting design, whereas their specific application to direct AChE/PAS-binding peptides remains comparatively underdeveloped. The sequence of engineering and evidence-gating steps is summarized in Figure 4.

### 8.1. Why Native Peptides Often Fail as Multifunctional AChE Modulators

Native and linear peptides commonly exhibit pharmacological liabilities including rapid proteolytic degradation, short systemic half-life, poor oral bioavailability, limited membrane permeability, nonspecific tissue distribution, and, for CNS applications, inefficient brain exposure [14,41]. Peptide therapeutics may also undergo intracellular degradation after uptake, while chemical conjugation can substantially alter the physicochemical and pharmacological properties of the parent molecule [41].

These limitations are particularly relevant to CNS-directed multifunctional peptides because pharmacological activity requires not only preservation of the intended molecular recognition motif but also sufficient exposure at the relevant anatomical site. For an AChE-centred construct, this may require simultaneous access to the enzyme’s catalytic gorge and PAS, while a second pharmacological activity may need to be maintained in the same molecular species.

Consequently, engineering should be considered a mechanism-preserving optimization process. Cyclization, stereochemical modification, retro-inverso transformation, backbone modification, incorporation of non-natural amino acids, lipidation, PEGylation, and peptide–drug or peptide–shuttle conjugation can each alter affinity, selectivity, proteolytic stability, solubility, pharmacokinetics, tissue distribution, and cellular uptake [41,79]. Each modified construct should therefore be treated as a new pharmacological entity requiring independent biochemical and pharmacokinetic characterization.

### 8.2. Cyclization

Cyclization is a well-established strategy for restricting peptide conformational flexibility and improving resistance to proteolysis. Conformational constraint can also increase binding affinity when the constrained structure approximates the bioactive conformation of the parent peptide [80,81]. However, these effects are highly sequence- and target-dependent, and cyclization can equally reduce activity if the required recognition geometry is disrupted.

For multifunctional AChE peptide design, cyclization is therefore best regarded as a prospective strategy for stabilizing and pre-organizing target-recognition elements, rather than as an established method for producing PAS-selective AChE peptides. Its potential advantages should be assessed by comparing the linear and cyclic forms of the same sequence with respect to AChE inhibition, PAS interaction, Aβ modulation, proteolytic stability, and cellular activity.

Cyclization should also not be assumed to improve BBB penetration. CNS exposure depends on multiple physicochemical and biological determinants, and increased conformational stability does not necessarily translate into increased brain permeability [82].

### 8.3. D-Amino-Acid Substitution

Replacement of selected L-amino acids with their D-enantiomers is widely used to increase resistance to proteolytic degradation. Depending on the sequence and structural requirements of the target, this approach can substantially extend the biological lifetime of peptide sequences while preserving the spatial presentation of selected side chains [77,83].

The RI-OR2 peptide provides a particularly relevant example in the amyloid field. RI-OR2 was designed as a retro-inverso derivative of the Aβ oligomerization inhibitor OR2 and was highly resistant to degradation in human plasma and brain extracts while retaining the ability to inhibit Aβ oligomer and fibril formation [84]. Similarly, arginine-rich D-enantiomeric peptides such as D3 and RD2 have been investigated as proteolytically stable Aβ-binding peptides.

These studies establish the feasibility of D-peptide engineering for amyloid-targeting applications, but they should not be interpreted as evidence that the same modifications preserve direct AChE/PAS recognition. For an AChE-targeting sequence, stereochemical substitutions must therefore be evaluated experimentally because the contribution of backbone geometry and individual side chains to AChE recognition may differ substantially from that in Aβ binding.

### 8.4. Retro-Inverso Design

Retro-inverso design combines sequence reversal with replacement of L-amino acids by D-amino acids, generating a peptide in which the side-chain topology can approximate that of the parent L-peptide while substantially altering the peptide backbone and proteolytic susceptibility [41].

RI-OR2 represents a well-characterized example. Compared with its parent OR2 sequence, RI-OR2 showed markedly increased proteolytic stability and retained activity against Aβ oligomer and fibril formation [84]. Importantly, subsequent attachment of a retro-inverso TAT sequence produced RI-OR2-TAT, which crossed the BBB in an amyloid precursor protein/presenilin-1 (APP/PS1) mouse model and was associated with reductions in Aβ oligomers, amyloid plaque burden, activated microglia, and oxidative damage [85]. This work illustrates a broader engineering principle: molecular recognition, proteolytic stability, and CNS delivery can be optimized sequentially within the same peptide platform.

Nevertheless, retro-inverso design should not be assumed to preserve every peptide–protein interaction. The approach is most appropriate when recognition is dominated by side-chain topology rather than by precise backbone hydrogen-bonding interactions. Its applicability to AChE/PAS-binding sequences therefore requires direct structural and functional validation.

### 8.5. N-Methylation and Non-Natural Amino Acids

Backbone N-methylation modifies peptide hydrogen-bonding capacity, conformational preferences, lipophilicity, and proteolytic stability and has become an important strategy in the development of more drug-like peptide molecules. Non-natural amino acids provide an additional means of modifying steric properties, conformational restriction, hydrophobicity, charge, and metabolic stability [41].

These modifications are particularly attractive when a short peptide contains a defined pharmacophore but exhibits inadequate pharmacokinetic or physicochemical properties. Each residue should therefore be modified selectively and evaluated using matched structure–activity and pharmacokinetic studies.

N-methylation has also been explored in amyloid aggregation inhibitors, including β-sheet-breaking strategies. N-methylated peptides derived from Aβ recognition sequences have been shown to inhibit Aβ aggregation and toxicity, demonstrating how backbone modification can interfere with pathological peptide self-assembly [83,84]. Such examples are mechanistically informative for Aβ-targeting design, but they should not be presented as direct evidence for N-methylated AChE/PAS modulators unless such molecules have been experimentally tested against AChE.

### 8.6. Bifunctional Constructs, Linkers, and Targeting Modules

A particularly attractive direction for multifunctional peptide development is the construction of a single molecule containing two independently optimized functional elements. In principle, one domain could provide AChE/PAS recognition, whereas another could recognize Aβ or provide an independent neuroprotective, antioxidant, metal-chelating, or neurotrophic function.

The success of such constructs depends critically on linker architecture. Linker length, flexibility, charge, and hydrophilicity can determine whether the two pharmacophores remain simultaneously accessible or interfere sterically with one another. Accordingly, simply connecting two active peptides does not establish a functional multifunctional ligand.

An instructive example of modular engineering is RI-OR2-TAT, in which the Aβ-binding/aggregation-inhibitory peptide was combined with a cell-penetrating sequence to improve cellular and brain access [86]. Subsequent incorporation of RI-OR2-TAT into liposomal nanoparticles produced peptide inhibitor nanoparticles (PINPs), which displayed markedly enhanced apparent potency against Aβ aggregation and were able to cross an in vitro BBB model and enter the brain in experimental animals [87]. These studies illustrate the value of modular peptide engineering, although the resulting constructs remain Aβ-directed rather than AChE-directed.

For future AChE-centred constructs, the same design logic could be applied by combining a validated AChE/PAS-binding pharmacophore with an independently validated second module. Such a construct should then be demonstrated to retain both activities rather than assuming that the parent activities remain intact after conjugation.

### 8.7. Lipidation and PEGylation

Lipidation and PEGylation are established approaches for modifying peptide pharmacokinetics. Lipidation can promote association with plasma proteins, particularly albumin, and may prolong systemic exposure, whereas PEGylation can increase hydrodynamic size and reduce renal clearance, thereby extending circulation time [85,88].

However, these benefits may be accompanied by trade-offs. In CNS applications, prolonged systemic exposure does not necessarily translate into increased brain exposure, and PEGylation may be disadvantageous if the modified peptide cannot efficiently reach the CNS [14,88].

This distinction is particularly important for multifunctional AChE peptides. Therefore, lipidated or PEGylated constructs should be evaluated using matched measurements of plasma stability, intact-peptide exposure, brain concentration, AChE target engagement, and secondary pharmacology.

### 8.8. BBB-Shuttle and Cell-Penetrating Peptide Conjugation

CNS delivery represents a major bottleneck for peptide therapeutics. Cell-penetrating peptides (CPPs) and dedicated BBB-shuttle peptides have therefore been investigated as means of enhancing cellular uptake or transporting molecular cargo across the BBB. Examples include TAT-derived sequences and receptor-mediated transcytosis approaches [82,89,90].

The RI-OR2-TAT system demonstrates the potential of this strategy in AD models. Peripheral administration of RI-OR2-TAT resulted in detectable brain localization and reduced several amyloid-associated pathological endpoints in APP/PS1 mice [86]. However, CPP-mediated cellular uptake should not be equated with efficient parenchymal delivery or therapeutic brain exposure.

Accordingly, a BBB shuttle should be regarded as a delivery module rather than a pharmacological mechanism. For multifunctional AChE constructs, successful design would require demonstration that the intact conjugate reaches the CNS, remains chemically intact, engages AChE, and preserves the intended second activity.

### 8.9. Peptidomimetics

Peptidomimetics seek to retain the key pharmacophoric features of an active peptide while replacing metabolically or physicochemically problematic elements with non-peptidic or conformationally constrained structures. This strategy can reduce proteolytic susceptibility and improve pharmacokinetic properties while maintaining recognition of the biological target [78].

For AChE-centred multifunctional design, peptidomimetics are particularly attractive when a short peptide sequence contains a well-defined recognition motif but the complete peptide has inadequate CNS exposure or metabolic stability. Rather than preserving the entire peptide sequence, the minimal structural determinants responsible for AChE/PAS recognition could be identified and progressively replaced with non-peptidic elements.

The ultimate objective would be to obtain a molecule that retains the extended molecular recognition achievable with peptide scaffolds while acquiring some of the pharmacokinetic advantages of small molecules. This approach is conceptually consistent with the broader development of peptide-derived and peptidomimetic ligands, but specific peptide-derived AChE/PAS peptidomimetics require direct experimental validation [91].

### 8.10. Design Principles and Experimental Validation

Molecular engineering should therefore be viewed as an iterative optimization process rather than a collection of independent chemical modifications. A useful development sequence would include:

1. Identification of the minimal AChE/PAS recognition motif;
2. Structural characterization of its interaction with AChE;
3. Optimization of proteolytic stability and conformational properties;
4. Incorporation of a second independently validated pharmacological function;
5. Optimization of CNS delivery;
6. Determination of intact-peptide pharmacokinetics and brain exposure; and
7. Demonstration of target engagement and biological activity in disease-relevant models.

For each engineering step, the modified peptide should be compared directly with the parent sequence. At minimum, studies should distinguish between biochemical AChE inhibition, PAS interaction, Aβ modulation, cellular activity, plasma stability, brain exposure, and in vivo target engagement.

This framework is particularly important because improved stability, increased cellular uptake, or enhanced cognitive performance does not by itself demonstrate multifunctional AChE activity. A peptide should be classified as a multifunctional AChE modulator only when direct AChE/PAS engagement and the independently intended second mechanism have both been experimentally demonstrated.

## 9. Blood–Brain Barrier Delivery and CNS Pharmacology of Multifunctional AChE Modulators

**Figure 5:**
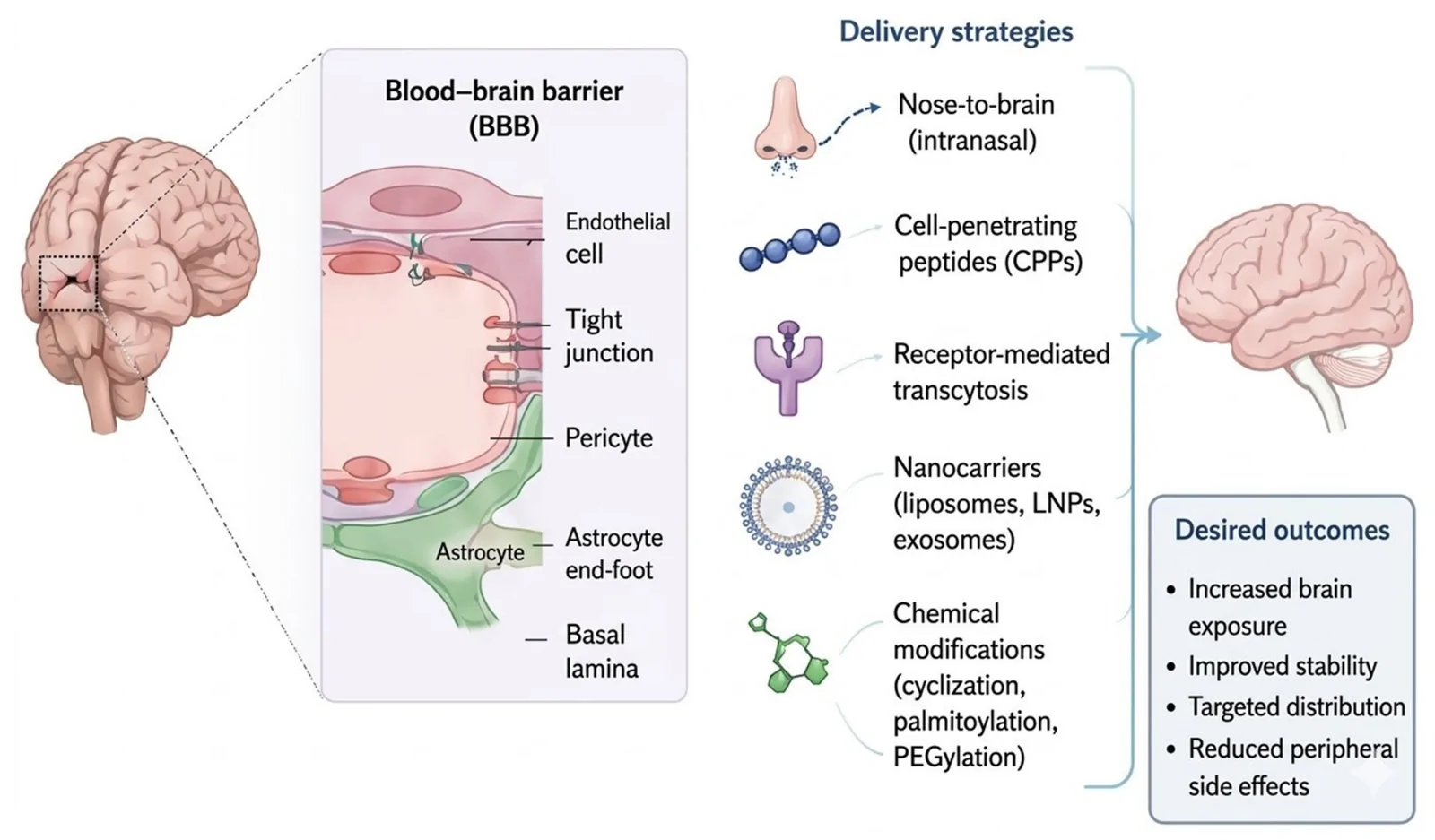
Strategies for enabling peptide delivery to the CNS, including receptor-mediated transport, cell-penetrating peptides, nanocarriers, chemical modification, and nose-to-brain administration. The delivery concepts are consistent with current reviews of intranasal and nanocarrier-based CNS delivery.

Figure 5 presents the mechanistic or translational relationships described in this section.

The BBB represents a major pharmacological determinant of the success of CNS-directed multifunctional modulators of AChE. A peptide may display high biochemical affinity for AChE and simultaneously interfere with amyloid-β (Aβ) aggregation, yet fail therapeutically if the intact active species does not reach the brain at sufficient concentrations and for a sufficient duration to engage the intended targets.

This issue is particularly important for peptides because molecular size, charge, hydrophilicity, conformational properties, proteolytic stability, and interactions with plasma proteins can strongly influence their distribution and clearance. Consequently, successful CNS delivery should be evaluated in terms of quantitative brain exposure, preferably including the unbound or pharmacologically available fraction, and pharmacologically relevant target engagement, rather than inferred from physicochemical properties, brain/plasma ratios, or peripheral pharmacodynamic effects alone [91,92].

### 9.1. BBB-Shuttle Peptides

Receptor-mediated transcytosis (RMT) is one of the principal strategies investigated for transporting macromolecules across the BBB. In this approach, a therapeutic peptide or protein is linked to a ligand or antibody-derived targeting element capable of interacting with a BBB-expressed receptor, such as the transferrin or insulin receptor.

This approach is sometimes described as a “Trojan Horse” strategy; however, receptor-mediated transport should be understood mechanistically rather than as a general guarantee of efficient brain delivery. Transport efficiency depends on receptor abundance, ligand affinity, valency, intracellular trafficking, endothelial recycling, and the balance between productive transcytosis and intracellular degradation [93,94].

For multifunctional AChE peptides, BBB shuttles could theoretically be incorporated as a third functional module alongside an AChE/PAS-binding domain and a second therapeutic pharmacophore. Peptide-based BBB shuttles and peptide–drug conjugates have been investigated using both receptor-mediated and cell-penetrating mechanisms, but their efficiency and translational performance remain highly construct-dependent [89,90,95].

Potential limitations include receptor-specific saturation, nonlinear pharmacokinetics, peripheral tissue distribution, intracellular sequestration, and possible alteration in the biological activity of the therapeutic cargo. Consequently, the addition of a BBB shuttle should be evaluated as a new molecular construct rather than assumed to preserve the properties of the parent peptide [93].

### 9.2. Nanocarriers

Nanocarrier systems provide an alternative approach to improving CNS exposure of peptide therapeutics. These systems can protect peptide cargo from degradation, modify pharmacokinetics, facilitate cellular uptake, and, when appropriately engineered, exploit endogenous transport pathways at the BBB [94].

Recent work emphasizes the potential of nanocarriers for CNS delivery while also highlighting substantial challenges related to formulation reproducibility, biodistribution, stability, toxicity, and the mechanisms governing BBB transport [35,94]. Peptides can additionally be incorporated into or displayed on nanocarriers as targeting ligands, thereby combining molecular recognition with a drug-delivery function [95].

For multifunctional AChE modulators, formulation must preserve the activity of the intact peptide or other pharmacologically active molecular species. Relevant parameters therefore include encapsulation efficiency, peptide integrity, release kinetics, particle stability, brain distribution, and retention of both AChE/PAS activity and the secondary pharmacological function.

Nanocarrier-mediated delivery should consequently not be interpreted as evidence of successful CNS pharmacology on the basis of brain-associated fluorescence, total tissue concentration, or nanoparticle localization alone. Measurements of total brain concentration can be particularly misleading when substantial fractions of the compound remain associated with vascular, endothelial, intracellular, or other non-target compartments [92].

### 9.3. Intranasal Delivery

Intranasal administration is an attractive non-invasive approach for CNS-directed delivery of peptides and other macromolecules. Depending on molecular and formulation properties, intranasally administered compounds may reach the CNS through a combination of systemic absorption and pathways associated with the olfactory and trigeminal regions [92,94].

However, the term “BBB bypass” should be used cautiously. Brain exposure is influenced by peptide molecular size, charge, lipophilicity, enzymatic stability, formulation composition, nasal residence time, mucociliary clearance, dosing volume, and species-specific nasal anatomy [92,94].

For multifunctional AChE peptides, intranasal administration may therefore represent a useful delivery strategy, but its success must be demonstrated quantitatively. Demonstration of AChE target engagement or a corresponding pharmacodynamic effect would provide substantially stronger evidence than measurements of total peptide-associated signal [92,94].

### 9.4. CNS Target Engagement

The ultimate criterion for successful CNS delivery is not simply the presence of a compound in the brain but pharmacologically meaningful target engagement. CNS drug-development frameworks increasingly emphasize the integration of pharmacokinetics, brain distribution, target engagement, pharmacodynamic biomarkers, and efficacy rather than relying on brain penetration measurements alone [76,91,96].

First, exposure of the intact active species should be established in the relevant CNS compartment. Second, AChE engagement should be demonstrated by biochemical or pharmacodynamic evidence, such as inhibition of catalytic activity or experimentally supported interaction with the PAS. Third, where Aβ modulation is part of the proposed mechanism, an independent measure of Aβ binding, aggregation, oligomerization, or pathology should be obtained.

This distinction is critical because brain exposure is not equivalent to target engagement, and target engagement at one component does not establish simultaneous engagement of all intended targets. Moreover, total brain concentration does not necessarily reflect the concentration available at the pharmacological target; contemporary CNS pharmacology therefore places particular emphasis on unbound brain concentrations and their relationship to pharmacological activity [92].

Accordingly, CNS development of multifunctional AChE modulators should incorporate a pharmacological chain of evidence linking:

administration → intact active species exposure → brain distribution → AChE/PAS engagement → secondary target engagement → downstream pharmacodynamic effect → disease-relevant phenotype.

This framework provides a more rigorous basis for evaluating CNS peptide candidates than reliance on behavioural efficacy, peripheral pharmacodynamic effects, or total brain-associated drug measurements alone. Where feasible, molecular imaging and other target-engagement approaches can further strengthen the translational chain by providing evidence of target occupancy or pharmacological activity in the living brain [76,96].

Importantly, this framework also implies that BBB engineering should not be considered successful merely because it increases brain-associated drug levels. For multifunctional AChE modulators, the relevant endpoint is functional delivery of the intact molecular species to the appropriate CNS compartment at concentrations sufficient to engage AChE/PAS and the independently intended secondary target.

## 10. Current Advances in Preclinical Evidence: From Biochemical Activity to Disease-Relevant Efficacy

**Figure 6:**
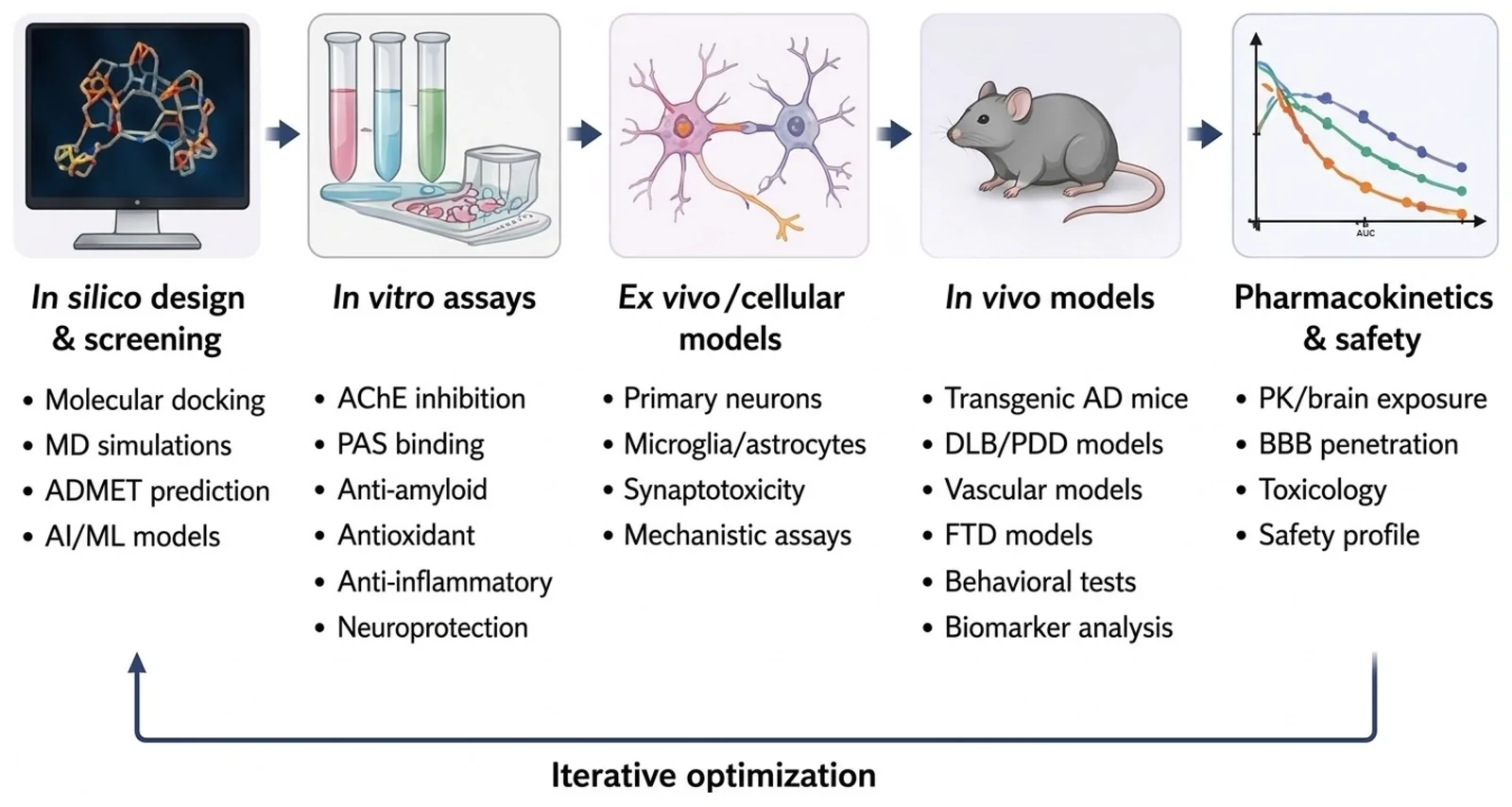
Proposed preclinical evaluation cascade for multifunctional AChE peptide modulators: in silico prioritization, biochemical and cellular testing, in vivo efficacy, pharmacokinetics/safety, and iterative optimization.

Figure 6 presents the mechanistic or translational relationships described in this section.

Preclinical evidence for peptides and peptide analogues proposed as multifunctional modulators of AChE should be evaluated along a stratified translational evidence chain rather than as a single measure of “multifunctionality.” This chain extends from biochemical target engagement, through cellular mechanism and functional activity, molecular stability and pharmacokinetics, CNS exposure, in vivo pharmacodynamics, and finally disease-relevant efficacy.

At the biochemical level, the primary question is whether a peptide or analogue directly interacts with AChE and alters its function. For candidate molecules proposed to act at the AChE–Aβ/PAS interface, this should ideally include experimental evidence of binding or inhibition together with structural or mechanistic information identifying interaction with the catalytic gorge, PAS, or both. The multifunctional role of AChE in amyloid-associated pathology, including its interaction with Aβ through the peripheral region of the enzyme, provides a mechanistic rationale for this type of target-based design [10,11,12,22,33].

The next level concerns cellular mechanism and functional activity. Here, the relevant question is whether the biochemical interaction produces a reproducible cellular phenotype and whether that phenotype can be mechanistically attributed to AChE modulation or to an independently demonstrated secondary mechanism. This distinction is particularly relevant for peptides such as PACAP, for which receptor-mediated neuroprotective effects against Aβ toxicity have been demonstrated in cellular and animal models without establishing direct AChE inhibition [63,64].

Molecular stability and pharmacokinetics constitute an additional translational filter. For peptides, susceptibility to proteolysis, limited systemic half-life, poor membrane permeability, and rapid clearance may prevent an otherwise active molecule from reaching its intended target at pharmacologically relevant concentrations [13,41]. Chemical strategies such as retro-inverso design can substantially improve proteolytic stability; for example, RI-OR2 was developed as a proteolytically stable inhibitor of Aβ oligomerization while retaining anti-amyloid activity [41].

CNS exposure represents a further critical step. Wherever possible, studies should distinguish total brain-associated material from exposure to the intact pharmacologically active species and should relate exposure to concentrations capable of engaging the proposed target. The BBB represents a major determinant of CNS pharmacology, and receptor-mediated transport and peptide-based delivery strategies may improve brain access while introducing additional variables related to transport efficiency, degradation, and distribution [93,94].

The subsequent level is in vivo pharmacodynamics and target engagement. Stronger evidence is obtained when CNS exposure can be linked to measurable modulation of AChE activity, AChE-associated signalling, Aβ-related processes, or another explicitly defined secondary target. This distinction is particularly important for multifunctional candidates, for which a behavioural phenotype may arise from several pharmacological mechanisms operating simultaneously [20,23].

Finally, disease-relevant efficacy should be evaluated using endpoints appropriate to the pathological context under investigation. Cholinergic dysfunction provides a strong therapeutic rationale in Alzheimer’s disease and Lewy body disorders [1,2], whereas the contribution of cholinergic mechanisms and amyloid pathology is more heterogeneous across vascular and frontotemporal dementias [2,81,97].

The maturity of the evidence therefore differs substantially among peptide classes. Rather than repeating the same validation hierarchy across sections, the present review applies it at the candidate level: each mechanistic claim is linked to the strongest available compound-specific evidence, while gaps in direct target engagement, selectivity, CNS exposure, or clinical efficacy are stated explicitly [98].

For this reason, evidence maturity should remain explicit throughout the review. A peptide showing activity in several of these domains may be a promising multifunctional candidate, but the mechanistic strength of each individual claim must remain proportional to the underlying evidence [98].

Overall, the current literature supports a rational design and evaluation framework for multifunctional AChE-related peptides, rather than a large body of already validated multifunctional AChE modulators. Future candidates should be advanced through an integrated sequence of biochemical, cellular, pharmacokinetic, CNS, pharmacodynamic, and disease-model validation rather than being classified as multifunctional solely on the basis of phenotypic or multi-assay activity (Table 6).

**Table 6:** Stratified preclinical evidence chain for multifunctional AChE modulators.

Preclinical Level	Key Question	Preferred Evidence
Biochemical target engagement	Does the molecule directly modulate AChE or interact with the AChE–Aβ/PAS interface?	Enzymatic kinetics (e.g., IC_50_, K_i_), direct binding assays, and structural or biophysical evidence demonstrating target interaction [21,33,34,38,99].
Cellular mechanism	Does the proposed target modulation produce the expected cellular phenotype?	Disease-relevant neuronal, synaptic, or glial models with mechanistically matched functional and molecular readouts [20,21,23,63,65,66].
Stability/metabolism	Does the active peptide remain sufficiently intact to reach and engage the intended target?	Plasma and tissue stability studies, proteolytic degradation assays, metabolite characterization, and identification of the intact active species [13,15,41,84,100].
CNS pharmacokinetics	Does the intact active species reach the brain at pharmacologically relevant exposure?	Quantitative brain exposure, brain-to-plasma relationships, identification of intact versus degraded species, and exposure–time profiles; where feasible, assessment of unbound brain exposure [91,92,93,94,101].
In vivo pharmacodynamics/target engagement	Does CNS exposure produce measurable modulation of AChE or the proposed secondary pathway?	Direct ex vivo or in vivo AChE assays, target-engagement measurements, pathway biomarkers, and exposure–response relationships [20,21,23,76,92,102].
Disease-relevant efficacy	Does the proposed mechanism translate into disease-relevant molecular, pathological, synaptic, or behavioural outcomes?	Cognitive and behavioural endpoints, amyloid or other disease-related pathology, synaptic and inflammatory markers, together with mechanistically matched pharmacodynamic evidence [49,50,51,52,53,54,55,56,57,58,59,60,61,62,63,66,85,86].
Translation readiness	Is the candidate sufficiently characterized to justify further preclinical or clinical development?	Defined dose–exposure–response relationships, preliminary safety and tolerability, formulation feasibility, manufacturability, reproducibility, and appropriate pharmacokinetic/pharmacodynamic margins [17,30,41,103,104].

## 11. Clinical Evidence and Therapeutic Perspectives in AD and Other Dementias

Clinical evidence for peptides relevant to AChE-associated dementia remains limited and heterogeneous. Cerebrolysin provides human evidence for a peptide-derived neurotrophic preparation that has been evaluated in randomized clinical studies in Alzheimer’s disease and vascular dementia, with some studies reporting improvements in cognitive, global, or functional outcomes [57,60,61,62].

Davunetide (NAPVSIPQ) illustrates the difficulty of translating a mechanistically defined neuroprotective strategy into consistent clinical benefit. In a double-blind, placebo-controlled, ascending-dose study of 144 individuals with mild cognitive impairment, intranasal davunetide was generally safe and well tolerated, but the prespecified composite cognitive memory outcome did not show a statistically significant treatment difference, although signals of potential efficacy were observed in selected measures of memory and attention [102].

The clinical history of davunetide also illustrates the importance of testing a mechanistically matched therapeutic hypothesis. Its subsequent evaluation in progressive supranuclear palsy did not demonstrate a clinically meaningful disease-modifying effect: in a randomized phase 2/3 trial involving 313 participants, davunetide did not differ from placebo on the primary measures of disease progression [56]. Davunetide is therefore best regarded as a clinically investigated neuroprotective peptide with an indirect relationship to cholinergic mechanisms rather than as an AChE-directed therapeutic candidate.

The negative clinical result is informative for the present review because it demonstrates that mechanistic plausibility is only one component of translational success. It does not, however, justify the converse conclusion that multifunctional AChE peptides will fail. Rather, future constructs must demonstrate the full pharmacological chain—intact-species exposure, AChE/PAS target engagement, engagement of the secondary mechanism, exposure–response relationships, and disease-relevant pharmacodynamic effects—before clinical efficacy can be meaningfully interpreted [20,21,23,75,76].

For future multifunctional AChE-related peptides, clinical development should be biomarker-guided and mechanism-driven. Early clinical studies should define the intended molecular mechanism, characterize systemic and CNS exposure to the intact active species where technically feasible, and incorporate pharmacodynamic or target-engagement biomarkers that can demonstrate biological activity in the relevant compartment. In CNS drug development, mechanistic biomarkers are particularly valuable because they can establish whether a candidate has produced pharmacologically driven biological activity in the brain before large clinical efficacy trials are undertaken [75,98,105].

For an AChE-related multifunctional peptide, a rational clinical development programme should therefore distinguish at least four levels of evidence: (i) exposure to the active species, (ii) molecular target engagement or pharmacodynamic modulation, (iii) downstream disease-relevant biological effects, and (iv) clinical cognitive or functional benefit. Such a strategy would reduce the risk of interpreting an absence of clinical efficacy as a failure of the molecular hypothesis when insufficient CNS exposure or target engagement has not been established [98,105].

Importantly, clinical efficacy should not be used as retrospective evidence of AChE engagement. Clinical improvement following a neuroprotective peptide preparation may result from multiple biological mechanisms, including modulation of trophic signalling, synaptic function, inflammation, oxidative stress, amyloid or tau-related processes, without requiring direct interaction with AChE.

Overall, the available clinical literature supports the feasibility of testing peptide-based therapeutics in dementia, but it does not yet establish a clinically validated class of multifunctional AChE-modulating peptides. Cerebrolysin demonstrates that peptide-derived neurotrophic preparations can be evaluated in AD and vascular dementia populations, whereas davunetide illustrates both the feasibility of clinical peptide development and the uncertainty involved in translating a mechanistically focused neuroprotective strategy into robust cognitive or disease-modifying efficacy [56,57,60,61,62,98].

## 12. Comparative Evaluation of Peptide and Peptide Analogues as Multifunctional Modulators

This section integrates the candidate-level evidence matrices to distinguish direct biochemical AChE/PAS findings from secondary mechanisms, CNS exposure, and clinical evidence before the comparative tables are presented. Table 7 provides the compound-specific evidence matrix.

Evidence-level descriptors used in the candidate matrices are operational rather than subjective. “Direct AChE/PAS evidence” requires compound-specific biochemical, binding, biophysical, structural, or mutational evidence of interaction; “secondary mechanism demonstrated” requires an independently measured disease-relevant mechanism in the same candidate; “clinical evidence” requires human intervention data with defined endpoints; and “not established” means that no qualifying evidence was identified in the narrative searches described in Section 2, not that absence of evidence has been proven by a systematic review. Because older literature and incomplete indexing can affect retrieval, negative classifications should therefore be interpreted as evidence gaps rather than evidence of absence. The class-level comparison is summarized in Table 8.

## 13. Therapeutic Perspectives: A Practical Translational Framework for Multifunctional AChE Modulators

**Figure 7:**
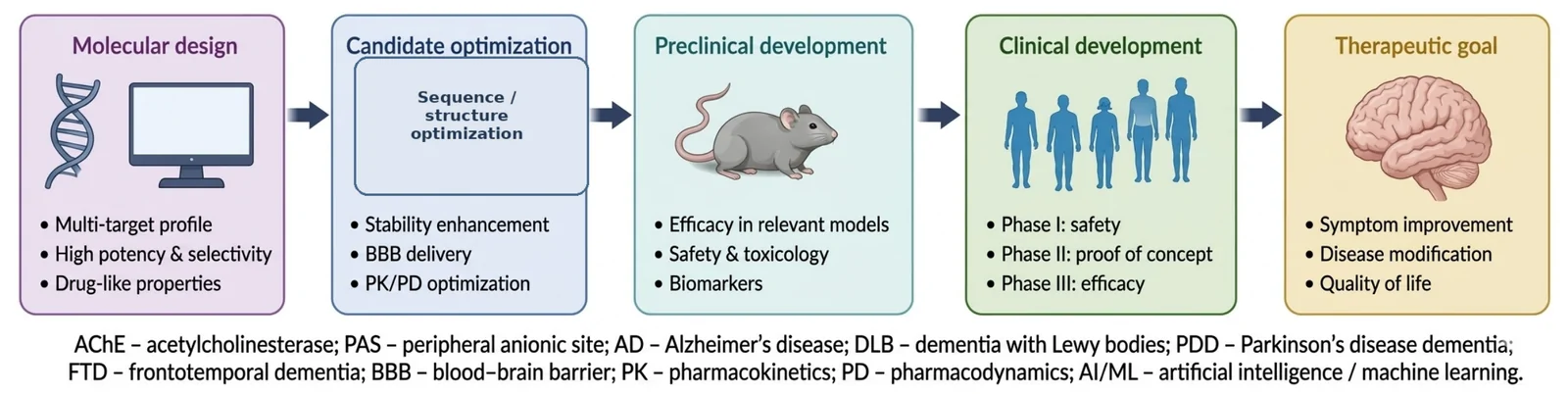
Proposed translational pathway from molecular design through candidate optimization and preclinical development to clinical testing and therapeutic goals. The pathway is a development framework rather than evidence that the complete chain has been demonstrated for current peptide candidates; progression requires independent validation at each stage.

**Figure 8:**
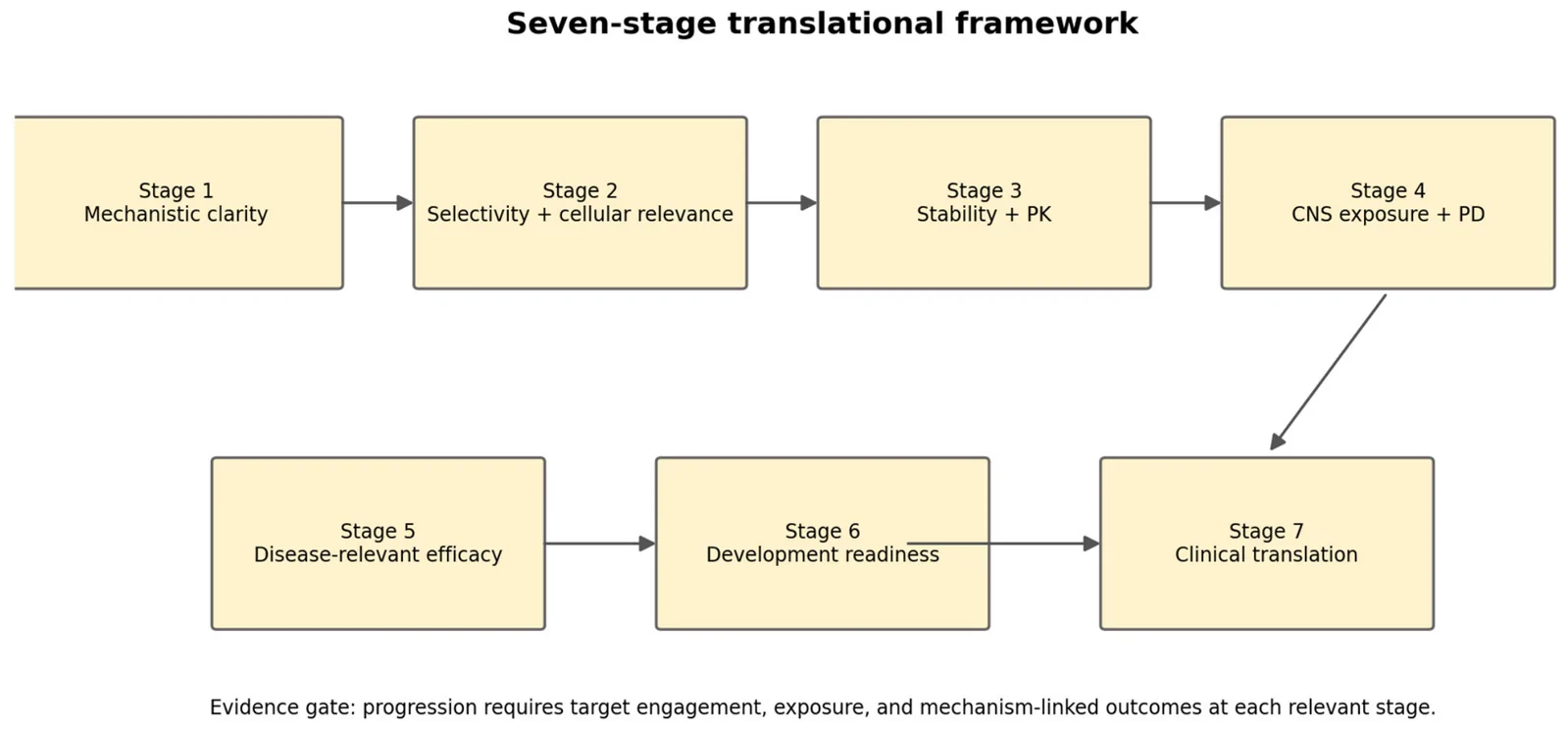
Seven-stage translational framework for multifunctional AChE-related peptide development, from mechanistic definition to biomarker-guided clinical translation. Progression between stages is evidence-gated and should require demonstration of the relevant target engagement and pharmacological chain.

The therapeutic perspective developed in this review is centred on a staged translational framework for the development of next-generation multifunctional AChE-related peptides. The framework reflects the current maturity of the evidence base: direct AChE inhibition can be quantified biochemically, whereas PAS engagement and proposed secondary functions require progressively more specific structural, biophysical, cellular, and in vivo validation [20,21,23,33,34,36,38,99] (Figure 7). The seven stages are also visualized in Figure 8.

Stage 1: Mechanistic clarity

The first step is to define each component of the proposed multifunctional mechanism independently. Direct AChE modulation should be demonstrated using purified-enzyme assays and, where appropriate, kinetic analyses that distinguish catalytic-site inhibition from other modes of interaction [2,33,34,38,99]. A proposed second function, such as Aβ recognition or inhibition of Aβ aggregation, should likewise be demonstrated using an independently validated assay [39,75,78,84,85,90,95].

Stage 2: Selectivity and cellular relevance

The second stage addresses pharmacological selectivity and biological relevance. This is particularly important because AChE and BChE have overlapping but non-identical substrate and inhibitor profiles, and BChE may itself become pharmacologically relevant during AD progression [2,98]. Whenever possible, pharmacological perturbation or genetic controls should be used to distinguish AChE-dependent effects from activity mediated by the proposed secondary mechanism [20,21,23,26,27,30].

Stage 3: Stability, metabolism, and pharmacokinetics

The third stage evaluates whether the molecular entity tested in vitro is also the biologically relevant species in vivo. Peptide degradation is a major determinant of pharmacological performance, and proteolysis may generate fragments with altered potency, selectivity, distribution, or mechanism of action [13,15,41]. Consequently, chemical stabilization should not automatically be regarded as pharmacologically neutral: cyclization, incorporation of D-amino acids, sequence modification, conjugation, or other structural changes may alter both pharmacokinetic properties and target recognition [41,80,81,83,84,100,107,108,109].

Stage 4: CNS exposure and pharmacodynamics

For CNS-directed development, demonstration of brain-associated material is necessary but not sufficient. The relevant question is whether the intact pharmacologically active species reaches the appropriate CNS compartment at concentrations compatible with its proposed mechanism [88,89,91,92,93,94,95,96,101]. Biomarker strategies that demonstrate pharmacological activity in the brain can substantially improve interpretation of early CNS development and reduce the risk of advancing compounds whose apparent exposure does not correspond to meaningful target engagement [20,21,23,75,102,105].

Stage 5: Disease-relevant efficacy

The fifth stage examines whether the validated mechanism translates into a disease-relevant phenotype. Improvements in cholinergic neurotransmission, for example, should be distinguished from evidence of modification of amyloid burden, neuroinflammation, synaptic dysfunction, tau pathology, or neurodegeneration [10,11,12,22,33,34,75,105]. Conversely, changes in pathological biomarkers should be interpreted together with pharmacodynamic and functional data rather than as surrogate evidence of clinical benefit in isolation [75,105].

Stage 6: Development readiness

Once mechanistic and translational activity has been established, development should address practical pharmaceutical and safety requirements. Nonclinical safety assessment should also consider peptide-specific issues such as metabolites, impurities, immunogenicity, and the consequences of non-proteogenic amino acids or other chemical modifications [41,91,100,107].

Stage 7: Clinical translation

The final stage is a biomarker-guided clinical development strategy in a population for which the proposed mechanism is biologically justified. In AD, for example, amyloid- and tau-related biomarkers can provide evidence concerning the underlying disease process and its modification, whereas cognitive and functional measures remain necessary to establish clinical benefit [75,105,110]. The clinical programme should therefore distinguish three levels of evidence: exposure to the active species, engagement or modulation of the proposed molecular target, and improvement in clinically meaningful outcomes. The decision points are summarized in Table 9.

Overall, this framework places mechanistic evidence before phenotypic interpretation and target engagement before claims of translational efficacy. For multifunctional AChE-related peptides, progression should therefore depend not on the number of biological activities attributed to a candidate, but on the strength of the causal evidence connecting molecular interaction, CNS exposure, pharmacodynamic modulation, and disease-relevant benefit [20,21,23,75,76,105].

## 14. Limitations and Unresolved Questions

The literature on multifunctional AChE-related peptides remains heterogeneous with respect to peptide sequence, molecular source, proposed mechanism, formulation, experimental system, disease model, and stage of development [13,41,98,99]. Many investigations report neuroprotective, antioxidant, anti-inflammatory, or anti-amyloid effects without demonstrating direct AChE engagement, whereas studies demonstrating biochemical AChE inhibition frequently provide limited information on peptide stability, brain exposure, or in vivo target engagement [38,41,54,63,65,98,99].

A related limitation is the frequent reliance on a single experimental level. Conversely, behavioural improvement in an animal model may indicate biological activity without identifying the molecular mechanism responsible for the effect [17,20,21,23,26,27,103,104]. Future studies should therefore incorporate orthogonal assays that connect biochemical interaction with cellular mechanism and, ultimately, in vivo pharmacodynamic evidence [20,21,23,30].

Peptide-specific pharmacokinetic liabilities represent an additional unresolved translational issue. Proteolytic degradation, short systemic half-life, limited membrane permeability, and uncertain CNS exposure can substantially reduce the relevance of promising in vitro findings [13,14,41,93,94,96]. Establishing the identity and pharmacological activity of the species that actually reaches the CNS is therefore an important prerequisite for interpreting in vivo efficacy [13,15,41,92,93].

Another unresolved question concerns the extent to which an AChE-centred multifunctional framework can be generalized beyond amyloid-associated disease. Cholinergic dysfunction is well established in dementia with Lewy bodies (DLB) and Parkinson’s disease dementia (PDD), providing a strong rationale for cholinergic symptomatic treatment [1,2,4,25,112]; however, the relevant pathological environment is dominated by α-synuclein-associated pathology and may involve variable contributions from amyloid and tau rather than the AChE–Aβ interaction alone [81].

The rationale is even more heterogeneous in vascular dementia (VaD) and frontotemporal dementia (FTD). In VaD, cholinergic dysfunction may coexist with vascular injury and, in some patients, concomitant AD pathology, making the biological justification for multi-target AChE modulation dependent on disease subtype and underlying pathology [2,31].

An additional unresolved issue is whether multifunctionality itself provides a therapeutic advantage over optimized single-target AChE inhibition. Combining AChE/PAS modulation with Aβ, tau, inflammatory, oxidative, or neurotrophic mechanisms may theoretically address multiple pathological processes, but additional molecular activities can also increase pharmacological complexity, off-target interactions, and uncertainty about the mechanism of clinical benefit [11,12,28,29,105].

Finally, the field lacks a sufficiently standardized definition of a “multifunctional AChE peptide.” A candidate should preferably be classified according to the level of evidence supporting each proposed function: direct AChE interaction, validated secondary target or pathological mechanism, CNS exposure, in vivo target engagement, and disease-relevant efficacy [20,21,23,41,105].

An additional limitation concerns the review process itself. Because this is a narrative rather than systematic review, selection bias cannot be excluded, the completeness of database retrieval cannot be guaranteed, and exact historical database run dates were not prospectively logged. We also did not apply a formal study-quality or risk-of-bias instrument to every included report. Accordingly, the synthesis should not be interpreted as an exhaustive quantitative estimate of the peptide–AChE literature. Instead, we prioritized mechanistically informative and quantitatively characterized studies and explicitly distinguish information that was unavailable from information that was not reported in the source study.

Taken together, the major unresolved question is not whether peptides can display multiple biological activities, but whether a mechanistically defined, CNS-exposed, pharmacodynamically active multifunctional AChE peptide can provide a clinically meaningful advantage over existing therapies. Addressing this question will require disease-specific target selection, rigorous target-engagement studies, characterization of the active molecular species, quantitative CNS pharmacology, and biomarker-guided clinical development rather than further expansion of multifunctional claims based primarily on isolated in vitro or behavioural observations [20,21,23,75,92,102,105].

## 15. Future Directions

In the near term, progress should focus on hypothesis-driven molecular engineering rather than on increasing the number of biological activities attributed to individual candidates. Each peptide should be designed around a clearly defined role for AChE, the PAS, or another biologically justified cholinergic mechanism, while each proposed secondary function should be independently measurable [11,12,25,28,29,41,99,105].

A second priority is the development of integrated CNS-delivery strategies. BBB-shuttle approaches, receptor-mediated transcytosis, cell-penetrating or carrier-assisted systems, lipid-based nanoparticles, and intranasal administration may improve CNS exposure, but none should be considered sufficient on the basis of delivery alone [35,92,93,94,95,96,111]. Future studies should quantify exposure of the intact active species in the relevant brain compartment and determine whether chemical modification or formulation preserves AChE/PAS interaction and the intended secondary pharmacology [13,41,91,100,107].

A third priority is computation-driven but experimentally interpretable design. Molecular docking, molecular dynamics, quantitative structure–activity relationships, machine learning, and generative models can be used to explore sequence–structure–activity relationships and prioritize candidates for synthesis and testing. Screening approaches, including high-throughput biochemical and cellular assays, can complement these computational methods when prioritizing candidates for synthesis and testing [114]. Experimental binding measurements, enzyme kinetics, selectivity profiling, stability studies, cellular assays, and CNS pharmacokinetic/target-engagement studies remain necessary to establish the biological validity of the predicted mechanism [20,21,23,25,38,41,99,105].

A fourth priority is mechanism- and biomarker-integrated clinical development. Future clinical trials of multifunctional AChE-related peptides should incorporate pharmacodynamic evidence of the intended molecular interaction wherever feasible and should prospectively connect exposure, target engagement, downstream biological effects, and clinical outcomes [20,21,23,75,105]. Biomarker-guided early-phase studies could consequently provide an important decision point before larger efficacy trials, allowing investigators to determine whether the candidate reaches the intended CNS compartment and produces the expected pharmacological effect [75,76,102,105].

Overall, the available evidence supports a rational design framework for multifunctional AChE-related peptides, but the direct peptide literature remains quantitatively limited. The strongest current evidence comprises short sequence-defined inhibitors with measurable IC_50_/K_i_ values and, in selected studies, docking-supported CAS/PAS interaction models; orthogonal PAS binding kinetics, AChE/BChE selectivity ratios, structural complexes, intact-species CNS exposure, and CNS target-engagement data remain important gaps. Future work should therefore prioritize experimentally testable sequence–structure–activity relationships rather than expanding the number of phenotypic activities attributed to largely indirect neuropeptides.

Finally, future programmes should adopt a progressive evidence-gating strategy in which advancement depends on predefined translational milestones. This approach would reduce the risk of advancing peptides on the basis of isolated biochemical or behavioural findings and would provide a more reliable bridge between molecular engineering, pharmacology, and clinically meaningful outcomes [17,20,21,23,103,104].

## 16. Discussion

Taken together, the evidence supports a distinction between the feasibility of individual peptide functions and the much stronger claim of a validated multifunctional AChE-directed therapeutic. The direct enzyme studies of short sequence-defined peptides, including the pea-derived QSQS, LQHNA, SQSRS, ETRSQ and PQDER series, demonstrate measurable AChE inhibition and provide kinetic and docking information [38,99]. However, the docking-based CAS/PAS assignments in these studies remain mechanistic hypotheses rather than orthogonal proof of PAS engagement. This contrasts with the amyloid-directed literature, where KLVFF established a well-defined Aβ self-recognition motif and RI-OR2 provided an example of improved proteolytic stability and anti-amyloid activity [39,41,41]. The two evidence streams are complementary, but they do not by themselves establish a single peptide that independently engages AChE/PAS and an amyloid mechanism.

The clinical literature reinforces this evidence hierarchy. Cerebrolysin has been evaluated in randomized studies in dementia, but its heterogeneous composition prevents attribution of a sequence-defined AChE mechanism [57,60,61,62]. Davunetide provides a more chemically defined neuroprotective example, yet its failure to meet primary endpoints in progressive supranuclear palsy illustrates that mechanistic plausibility and clinical feasibility do not guarantee disease-modifying efficacy [56]. These findings are consistent with broader multi-target-directed-ligand literature, which emphasizes that polypharmacology requires experimentally substantiated engagement of the intended targets rather than inference from a composite phenotype [28,29].

The central critical issue is therefore not the number of biological effects reported for a peptide, but whether the same intact molecular species reaches the relevant CNS compartment, engages AChE/PAS at pharmacologically relevant exposure, retains an independently validated secondary activity, and produces a mechanistically linked pharmacodynamic effect. This requirement is particularly important for peptide therapeutics because proteolysis, altered active species, limited oral bioavailability, and uncertain BBB penetration can disconnect in vitro activity from in vivo exposure [13,14,15,41,91,92,93,94]. Consequently, behavioural improvement or anti-amyloid activity should not be used retrospectively to infer direct AChE modulation.

The hypothesis stated in the Introduction was therefore only partially supported. The literature supports the feasibility of combining sequence-defined peptide recognition, direct AChE inhibition, amyloid-directed activity, and engineering strategies within a rational design framework; however, the reviewed evidence does not establish a candidate that has experimentally demonstrated the complete multifunctional chain or a clinically validated class of multifunctional AChE modulators. The principal research gap is the absence of orthogonal, compound-specific target-engagement studies that connect AChE/PAS binding with an independently demonstrated secondary mechanism and CNS pharmacology. Future studies should therefore prioritize matched biochemical, structural, pharmacokinetic, pharmacodynamic, and disease-model experiments rather than increasing the number of phenotypic activities attributed to a single construct.

## 17. Conclusions

Peptides and peptide analogues provide a flexible platform for investigating cholinergic and disease-associated mechanisms in dementia, but the current evidence does not establish a clinically validated class of multifunctional AChE modulators [41,98,99]. Direct AChE-inhibitory peptides demonstrate biochemical feasibility, while amyloid-directed and neuroprotective peptides provide complementary disease-relevant activities; however, these functions have generally been demonstrated in separate molecular entities or at different levels of evidence. The translational priority is therefore to develop experimentally validated constructs in which AChE/PAS engagement and a second therapeutic mechanism are independently demonstrated, followed by confirmation of intact-species CNS exposure, target engagement, pharmacodynamic activity, and disease-relevant efficacy [20,21,23,25,28,29,98,105]. The AChE-centred rationale is strongest in disorders with established cholinergic dysfunction, particularly Alzheimer’s disease and Lewy body disorders, whereas the appropriate secondary mechanism should be adapted to disease-specific pathology [1,2,4,112]. Overall, the field should be regarded as an emerging, experimentally testable design space rather than a clinically established therapeutic class; progress will depend on rigorous separation of direct and indirect mechanisms, orthogonal target-engagement studies, quantitative CNS pharmacology, and evidence-gated translational development.

## Figures and Tables

**Table 1:** Evidence categories used in the review.

Evidence Category	Operational Definition	Minimum Evidence Expected	Interpretation
Direct AChE evidence [20,21]	Experimental interaction with AChE and/or its peripheral anionic site (PAS) has been demonstrated.	Biochemical inhibition, binding, kinetic analysis, structural or biophysical evidence, or an orthogonal target-engagement assay.	Supports AChE as a demonstrated molecular target.
Multifunctional AChE–Aβ evidence [10,11,12,22,23]	The candidate experimentally affects the AChE–Aβ interaction or an AChE-associated amyloid process.	Aβ aggregation assay performed in the presence of AChE, interaction or competition studies, structural/biophysical evidence, or equivalent mechanistic validation.	Supports modulation of an AChE-associated amyloid mechanism.
Indirect cholinergic evidence [1,2,3,5,6,7,24,25]	A cholinergic phenotype is modified without demonstrated direct engagement of AChE.	Neuronal protection, neurotransmitter measurements, receptor-level effects, or behavioural/pharmacological phenotypes.	Indicates biological relevance to cholinergic pathways but does not establish AChE as the molecular target.
Clinical evidence [1,3,5,7]	The intervention has been evaluated in humans.	A randomized or otherwise appropriately designed human intervention study with predefined clinical or biomarker endpoints.	Provides evidence of clinical activity or efficacy, but does not by itself establish the molecular mechanism.

**Table 2:** Direct biochemical inhibition of AChE and the gap between enzyme activity and the experimentally established involvement of the PAS.

Peptide	Sequence	IC_50_	K_i_	Inhibition Mode	CAS/PAS Evidence
QSQS	QSQS	4.25 μM (1.60 μg/mL)	0.43 mM	Mixed	Docking: PAS + CAS
LQHNA	LQHNA	3.29 μM (1.62 μg/mL)	0.16 mM	Non-competitive	Docking: PAS + CAS
SQSRS	SQSRS	3.26 μM (1.54 μg/mL)	0.02 mM	Uncompetitive	Docking: PAS + CAS
ETRSQ	ETRSQ	3.00 μM (1.57 μg/mL)	0.06 mM	Non-competitive	Docking: PAS + CAS
PQDER	PQDER	2.78 μM (1.54 μg/mL)	0.08 mM	Non-competitive	Docking: PAS + CAS
KLPGF	KLPGF	1.09 μM	NR	Not resolved	Docking: peripheral + catalytic regions

**Table 3:** Evidence status of peptide scaffolds at the AChE–Aβ/PAS interface.

Peptide/Scaffold	Demonstrated Function	AChE/PAS Evidence	Interpretation
KLVFF-derived sequences	Aβ recognition and inhibition of amyloid fibril formation [39,40]	No direct AChE/PAS evidence established in the reviewed studies	Aβ-directed scaffold; not a validated AChE modulator
RI-OR2	Proteolytically stable Aβ oligomer/fibril inhibition [41]	No direct AChE/PAS evidence established	Useful stability/anti-amyloid platform for future bifunctional design
D3/RD2	Aβ binding and interference with β-structure/aggregation [42,43,44]	No direct AChE/PAS evidence established	Stereochemically stabilized anti-amyloid scaffolds
Bifunctional AChE–Aβ concept	Proposed combination of AChE/PAS engagement and Aβ recognition	Requires independent demonstration of both functions	Design hypothesis rather than validated class

**Table 4:** Neuropeptides and neuroprotective peptide preparations discussed in Section 6.

Neuropeptide/Preparation	Principal Mechanism or Biological Role	Relevance to Neurodegeneration
PACAP	Receptor-mediated signalling; neuroprotective and anti-amyloid effects in experimental models [63,64]	Indirect cholinergic relevance; no direct AChE/PAS engagement established
VIP	Receptor-mediated signalling; modulation of microglial/inflammatory responses and neuroprotection [65,66]	Indirect neuroprotective comparator
NPY	Neuropeptide-receptor signalling affecting neuronal circuits and stress responses [67,68,69]	Associated with cholinergic/network and neuroprotective effects; no direct AChE evidence
Galanin	Neuropeptide signalling with effects on neuronal survival and cholinergic circuitry [70,71]	Indirect cholinergic relevance
Substance P	Neurokinin-receptor signalling with inflammatory and neuronal effects [72,73,74]	Indirect inflammatory/cholinergic-network relevance
Humanin	Mitochondrial and neuronal-survival signalling [46,47,48]	Neuroprotective comparator; not an established AChE modulator
Cerebrolysin	Heterogeneous peptidergic neurotrophic preparation [57,58,59,60,61,62]	Clinical neuroprotective evidence but no sequence-defined AChE mechanism
Davunetide (NAPVSIPQ)	Microtubule/tau-directed neuroprotective activity [54,55,56]	Mechanistically defined indirect comparator
Semax	Anti-amyloid, metal-chelating and neuroprotective effects [49,50,51,52]	Indirect anti-amyloid/neuroprotective comparator
GK-2	Nerve growth factor-loop mimetic neurotrophic activity [53]	Indirect neurotrophic comparator

**Table 7:** Compound-specific evidence matrix for peptide candidates with reported AChE activity or AChE-directed evidence. “Not reported” indicates that the source study did not provide the parameter; “unavailable” indicates that the accessible source did not contain sufficient information to reconstruct the condition. Docking-based PAS assignment is explicitly distinguished from direct PAS-binding evidence.

Candidate/Sequence	Quantitative AChE Activity	Assay Conditions/Controls	Selectivity/Secondary Mechanism	Independent/Orthogonal Evidence and Classification
QSQS/QSQS	IC_50_ 4.25 μM (1.60 ± 0.05 μg/mL); K_i_ 0.43 mM; mixed inhibition [38]	Electric-eel AChE; Ellman assay; 0.1 M phosphate pH 7.4; 37 °C; n = 3 for kinetic data; blank without inhibitor [38]	BChE selectivity ratio: not reported; no independently validated secondary mechanism reported	Fluorescence/circular dichroism (CD) changes and docking to PAS/CAS. Docking is supportive, not direct PAS binding. Direct AChE inhibition demonstrated; PAS engagement putative [38]
LQHNA/LQHNA	IC_50_ 3.29 μM (1.62 ± 0.02 μg/mL); K_i_ 0.16 mM; non-competitive [38]	Same assay framework; kinetic measurements at 0.05 and 0.10 mM peptide [38]	BChE selectivity ratio: not reported; no independently validated secondary mechanism reported	Fluorescence/circular dichroism (CD) changes; docking to PAS/CAS; Q at penultimate N-terminal position associated with stronger docking in this series. Direct AChE inhibition demonstrated; PAS engagement putative [38]
SQSRS/SQSRS	IC_50_ ≈ 3.26 μM (1.54 μg/mL); K_i_ 0.02 mM; uncompetitive [38]	Same assay framework; kinetic measurements at 0.05 and 0.10 mM peptide [38]	BChE selectivity ratio: not reported; no independently validated secondary mechanism reported	Fluorescence/circular dichroism (CD) changes; docking to PAS/CAS. Direct AChE inhibition demonstrated; PAS engagement putative [38]
ETRSQ/ETRSQ	IC_50_ 3.00 μM (1.57 μg/mL); K_i_ 0.06 mM; non-competitive [38]	Same assay framework; kinetic measurements at 0.05 and 0.10 mM peptide [38]	BChE selectivity ratio: not reported; no independently validated secondary mechanism reported	Fluorescence/circular dichroism (CD) changes; docking to PAS/CAS. Direct AChE inhibition demonstrated; PAS engagement putative [38]
PQDER/PQDER	IC_50_ 2.78 μM (1.54 μg/mL); K_i_ 0.08 mM; non-competitive [38]	Same assay framework; kinetic measurements at 0.05 and 0.10 mM peptide [38]	BChE selectivity ratio: not reported; no independently validated secondary mechanism reported	Fluorescence/circular dichroism (CD) changes; docking to PAS/CAS. Direct AChE inhibition demonstrated; PAS engagement putative [38]
KLPGF/KLPGF	61.23 ± 4.73% AChE inhibition at 50 μg/mL; primary study did not report a directly measured IC_50_ in the accessible report. An IC_50_ of 1.09 μM is reported in the 2018 review [99,106].	AChE/BChE inhibition assay at 50 μg/mL; detailed assay conditions are not reported in the primary abstract; therefore unavailable information is not inferred [106]	BChE inhibition 3.29 ± 0.93% at 50 μg/mL; no formal selectivity ratio reported [106]	Molecular docking indicated contacts with catalytic/acylation and peripheral anionic regions; no independent binding kinetics reported. Direct AChE inhibition demonstrated; PAS interaction supported by docking [106]

**Table 8:** Candidate- and class-level evidence matrix for peptide candidates discussed in the review.

Candidate/Class	Direct AChE/PAS Evidence	Secondary Mechanism/Phenotype	CNS/In Vivo Evidence	Clinical Evidence	Classification
Natural AChE-inhibitory peptides	Reported for selected peptides [38,99]	Primarily AChE inhibition; secondary functions require separate validation	Compound-dependent; generally limited	Not established as a class	Direct AChE candidates
KLVFF-derived peptides	No direct AChE/PAS evidence was identified in the narrative searches; this should not be interpreted as a systematic finding of absence [39,40].	Aβ recognition/aggregation inhibition [39,40]	Construct-dependent	None established	Aβ-directed/indirect
RI-OR2 and related D-peptides	No direct AChE/PAS evidence was identified in the narrative searches; this should not be interpreted as a systematic finding of absence [41,86].	Aβ oligomer/fibril inhibition; proteolytic stability [41,86]	RI-OR2-TAT shows brain localization and anti-amyloid effects in AD models [86]	None established	Aβ-directed/indirect
PACAP/VIP/NPY/galanin/Substance P	No direct AChE/PAS evidence was identified in the narrative searches; this should not be interpreted as a systematic finding of absence.	Neuroprotective, trophic, inflammatory or cholinergic-network effects [63,64,65,66,67,68,69,70,71,72,73,74]	Predominantly preclinical; compound-dependent	Not established as AChE modulators	Indirect cholinergic-relevant
Humanin	No direct AChE/PAS evidence was identified in the narrative searches; this should not be interpreted as a systematic finding of absence [46,47,48].	Neuroprotection/mitochondrial and survival signalling	Predominantly preclinical	Not established	Indirect neuroprotective
Cerebrolysin	No direct AChE/PAS evidence was identified in the narrative searches; this should not be interpreted as a systematic finding of absence [57,58,59,60,61,62].	Neurotrophic/neuroprotective effects	Clinical studies in AD and VaD [59,60,61,62]	Human randomized studies; heterogeneous evidence [59,60,61,62]	Peptide-derived multimodal preparation
Davunetide (NAP)	No direct AChE/PAS evidence was identified in the narrative searches; this should not be interpreted as a systematic finding of absence [54,55,56].	Microtubule/tau-related neuroprotection	Intranasal; clinical development [55,56,102]	Clinical trials; no established efficacy	Indirect neuroprotective
Semax	No direct AChE/PAS evidence was identified in the narrative searches; this should not be interpreted as a systematic finding of absence [49,50,51,52].	Copper-dependent anti-amyloid/redox effects; neuroprotection [49,50,51,52]	Preclinical AD models [52]	No established AD efficacy	Indirect anti-amyloid/neuroprotective
GK-2	No direct AChE/PAS evidence was identified in the narrative searches; this should not be interpreted as a systematic finding of absence [53].	NGF-mimetic neurotrophic effects	Preclinical rat AD models [53]	None established	Indirect neurotrophic

**Table 9:** Translational decision points for progression of a multifunctional AChE-related peptide candidate.

Development Question	Evidence Required for Progression
What is the dominant multifunctional target?	An explicit molecular hypothesis supported by validated and, where possible, orthogonal assays for each proposed activity. The multifunctional profile should be experimentally resolved into individual mechanisms rather than inferred from a composite phenotype [20,21,23,105].
Is the interaction direct?	Biochemical kinetics together with structural or biophysical evidence where feasible. For AChE, evidence should distinguish catalytic-site inhibition from interaction with peripheral/extended binding regions such as the PAS. Computational docking alone should be considered hypothesis-generating rather than proof of direct interaction [5,6,7,20,21,23,25,33,105].
Is the peptide selective?	Comparative assessment against AChE, BChE, and relevant off-targets, with selectivity interpreted in the context of the intended therapeutic mechanism. This is particularly important because AChE and BChE have overlapping but distinct pharmacological profiles [2,98].
Is the peptide pharmacologically stable?	Plasma and tissue stability studies, identification of degradation products and metabolites, and confirmation of which species retains the relevant activity. Proteolysis can substantially alter peptide exposure and generate species with different pharmacological properties [13,15,41,100,107].
Does chemical stabilization preserve the intended pharmacology?	Direct comparison of the modified and parent peptide for AChE/PAS interaction and the proposed secondary activity. Cyclization, D-amino-acid substitution, retro-inverso design, conjugation, or other modifications should not be assumed to preserve the original target profile [79,84,91,100,107].
Does the active species reach the CNS?	Quantitative CNS pharmacokinetics demonstrating exposure of the intact active species or a defined active metabolite in the relevant compartment. Detection of total peptide-associated material in brain tissue is insufficient to establish pharmacologically relevant CNS exposure [35,82,92,93,94,95,96,111].
Is there in vivo target engagement?	A pharmacodynamic biomarker, direct target assay, occupancy measurement, or other validated readout demonstrably linked to exposure and the proposed mechanism. CNS exposure and target engagement should be treated as separate development milestones [20,21,23,76,102].
Does the mechanism translate to phenotype?	Mechanistically matched cellular and animal endpoints, with appropriate pharmacological or genetic controls to distinguish AChE-dependent effects from effects mediated by the proposed secondary mechanism [17,20,21,23,26,27,30,103,104].
Does the candidate modify disease biology or only symptoms?	Evidence should distinguish symptomatic cholinergic effects from changes in disease-associated processes such as amyloid pathology, tau pathology, neuroinflammation, synaptic dysfunction, or neurodegeneration. Behavioural improvement alone should not be interpreted as proof of disease modification [6,7,10,17,22,33,34,103,104,105].
Is efficacy reproducible?	Independent replication, complementary disease-relevant models, predefined endpoints, and consistency across molecular, pathological, pharmacodynamic, and functional readouts. Reproducibility should be demonstrated before progression to more resource-intensive studies [17,26,27,30,103,104].
Can the candidate be manufactured reproducibly?	Scalable synthesis, defined formulation, purity specifications, impurity and degradation control, analytical characterization, and an appropriate quality-control strategy. These considerations are particularly important for synthetic peptide therapeutics [13,15,41,91].
Can it be dosed safely?	Nonclinical toxicology, immunogenicity assessment, dose–response relationships, exposure margins, and characterization of relevant metabolites, degradation products, and impurities [41,91,100,107].
Can a clinical trial directly test the hypothesis?	A defined mechanism, measurable biomarker or pharmacodynamic endpoint, biologically justified patient population, appropriate dose/exposure range, and clinically meaningful cognitive and/or functional outcomes. Mechanistic biomarkers should be incorporated prospectively rather than used retrospectively to explain efficacy [20,21,23,75,105].
Is the proposed mechanism appropriate for the target disease?	The cholinergic and pathological rationale should be established for the selected clinical population. The strength of the AChE-based rationale differs across dementias and should not be extrapolated uniformly from AD to DLB, PDD, VaD, or FTD [1,2,3,4,24,25,112,113].

## Data Availability

No new data were created or analyzed in this study. Data sharing is not applicable to this article.

## Abbreviations

Aβ, amyloid-β; ACh, acetylcholine; AChE, acetylcholinesterase; BChE, butyrylcholinesterase; AD, Alzheimer’s disease; BBB, blood–brain barrier; CAS, catalytic active site; CD, circular dichroism; CNS, central nervous system; DLB, dementia with Lewy bodies; FTD, frontotemporal dementia; GK-2, synthetic nerve growth factor-loop mimetic; IC_50_, half-maximal inhibitory concentration; ITC, isothermal titration calorimetry; K_i_, inhibition constant; MTDL, multi-target-directed ligand; NAP, NAPVSIPQ/davunetide; NMR, nuclear magnetic resonance; NPY, neuropeptide Y; PACAP, pituitary adenylate cyclase-activating polypeptide; PAS, peripheral anionic site; PD, pharmacodynamics; PDD, Parkinson’s disease dementia; PK, pharmacokinetics; PSP, progressive supranuclear palsy; RI-OR2, retro-inverso OR2 peptide; SPR, surface plasmon resonance; VaD, vascular dementia; VIP, vasoactive intestinal peptide.
